# Genomics-driven breeding for local adaptation of durum wheat is enhanced by farmers’ traditional knowledge

**DOI:** 10.1073/pnas.2205774119

**Published:** 2023-03-27

**Authors:** Cherinet Alem Gesesse, Bogale Nigir, Kauê de Sousa, Luca Gianfranceschi, Guido Roberto Gallo, Jesse Poland, Yosef Gebrehawaryat Kidane, Ermias Abate Desta, Carlo Fadda, Mario Enrico Pè, Matteo Dell’Acqua

**Affiliations:** ^a^Center of Plant Sciences, Scuola Superiore Sant’Anna, Pisa 56127, Italy; ^b^Amhara Regional Agricultural Research Institute, Bahir Dar 6000, Ethiopia; ^c^Digital Inclusion, Bioversity International, Parc Scientifique Agropolis II, Montpellier 34397, France; ^d^Department of Agricultural Sciences, Inland Norway University of Applied Sciences, Hamar 2322, Norway; ^e^Department of Biosciences, University of Milan, Milan 20133, Italy; ^f^Center for Desert Agriculture, King Abdullah University of Science and Technology, Thuwal 23955-6900, Saudi Arabia; ^g^Biodiversity for Food and Agriculture, Bioversity International, Addis Ababa 1000, Ethiopia; and; ^h^Biodiversity for Food and Agriculture, Bioversity International, Nairobi 00621, Kenya

**Keywords:** crop breeding, genomic selection, multiparental populations, smallholder farming, *Triticum durum* Desf.

## Abstract

In the smallholder, low-input farming systems widespread in sub-Saharan Africa, farmers select and propagate crop varieties based on their traditional knowledge and experience. A data-driven integration of their knowledge into breeding pipelines may support the sustainable intensification of local farming. Here, we combine genomics with participatory research to tap into traditional knowledge in smallholder farming systems, using durum wheat (*Triticum durum* Desf.) in Ethiopia as a case study. We developed and genotyped a large multiparental population, called the Ethiopian NAM (EtNAM), that recombines an elite international breeding line with Ethiopian traditional varieties maintained by local farmers. A total of 1,200 EtNAM lines were evaluated for agronomic performance and farmers’ appreciation in three locations in Ethiopia, finding that women and men farmers could skillfully identify the worth of wheat genotypes and their potential for local adaptation. We then trained a genomic selection (GS) model using farmer appreciation scores and found that its prediction accuracy over grain yield (GY) was higher than that of a benchmark GS model trained on GY. Finally, we used forward genetics approaches to identify marker–trait associations for agronomic traits and farmer appreciation scores. We produced genetic maps for individual EtNAM families and used them to support the characterization of genomic loci of breeding relevance with pleiotropic effects on phenology, yield, and farmer preference. Our data show that farmers’ traditional knowledge can be integrated in genomics-driven breeding to support the selection of best allelic combinations for local adaptation.

## Significance

Smallholder farming systems support the livelihoods of estimated two billion people on the planet. They are often characterized by challenging environments critically exposed to the climate crisis, with limited access to inputs including improved seed technology. To achieve the sustainable intesification of smallholder farming systems, crop breeding needs to tailor varietal development to local farmer needs. Here, we devise a method to integrate wheat genomics with participatory varietal selection that allows to capture farmers’ traditional knowledge in a fully quantitative framework and inform breeding decisions. Our work demonstrates that cultural and natural agrobiodiversity can be leveraged together to increase the effectiveness of genomics-driven breeding toward varietal development for smallholder farming systems.

Crop production systems worldwide are expected to be negatively affected by the climate crisis (1). To achieve and maintain a sustainable and equitable food production in a changing climate, farming systems need to increase their resilience while reducing their reliance on external inputs (2). This is crucial in the highly heterogeneous smallholder farming systems that are widespread in emerging countries (3), where limited access to agronomic inputs, including irrigation, fertilizers, and seed technology, limits buffering capacity to external shocks. Smallholder farming is a pivot of global food security (4), and strains to the system threaten the livelihoods of the millions of people depending on it (5). Crop breeding can support their resilience by accelerating the development of crop genotypes with adaptation to local growing conditions and end-user preferences, reducing the need for chemical inputs and increasing the impact of varietal innovation (6).

Smallholder farmers often rely on traditional varieties which evolved at the crossroads between anthropic and natural selection. If characterized and made accessible to breeding, this agrobiodiversity can contribute with adaptation traits (7), lowering the need for external inputs while increasing resilience in challenging farming environments. Vast genetic agrobiodiversity is maintained by farmers in situ as well as collected in genebanks, and current genomics methods allow to efficiently screen it to identify allele pools that may contribute to crop improvement (8). The most promising genotypes can be exploited as they are or be piped in breeding programs in various crossing designs, including backcrosses (9) and multiparental populations (10, 11), creating favorable allelic combinations. When genotyped collections of germplasm are tested in multiple growing environments, genomic selection (GS) models can be trained to capture genotype x environment interactions and predict the performance of specific allelic combinations, further accelerating genetic gain (12, 13). Forward genetic approaches may complement GS to identify quantitative trait loci (QTL) for complex traits including adaptation and agronomic performance, providing breeders with genetic targets to further crop improvement (14).

Agrobiodiversity that is found in smallholder farming systems is both nature and culture. When farmers select and maintain specific crop varieties and therefore specific allelic combinations, they consider a combination of traits that include performance, adaptation, and use (15). To support and improve varietal development for smallholder farming systems, researchers and breeders need to understand how varieties and seed demands vary by types of farmers, how these differences are reflected in seed acquisition dynamics, and how seed production can meet end-user demands (16). Today, only about 40% of smallholder beneficiaries adopt new varieties developed by breeding, a gap that calls for a reconsideration of the objectives of crop improvement targeting these farmers (17). Participatory variety selection (PVS) can be used to directly involve farmers in the selection of genetic materials and better align breeding decisions to end-user needs (18). In Ethiopia, PVS approaches showed that local wheat smallholder farmers select genetic materials according to a clear hierarchy of traits and may prioritize adaptation over performance (19). Previous studies confirmed that the evaluation of wheat phenotypes given by local farmers is repeatable and genetically determined as any other measure of agronomic performance (15) and can be used to map wheat genetic loci associated with farmer preference (20). Farmer evaluations of crop performance, an expression of their traditional knowledge in regard to farming, can thus be harnessed in a quantitative way and used to inform varietal recommendation (21) and improve genetic gain in challenging farming systems with decentralized, on-farm evaluation of genetic materials (22). Yet, the understanding of the factors underlying varietal suitability to local farmers’ requirements remains a major challenge for breeding.

To effectively target local adaptation, breeding must fully embrace a data-driven approach considering both cultural and natural aspects of agrobiodiversity. Here, we use a large multiparental population of Ethiopian durum wheat lines developed from local landraces to show that farmers’ traditional knowledge may be fully integrated in genomics-driven breeding methods relying on GS and QTL mapping. We run a PVS experiment in collaboration with local men and women farmers evaluating 10,400 plots in three locations in Ethiopia, exploring their preference in relation to genotypic and phenotypic diversity of tested wheat lines. We break down farmers’ overall appreciation (OA) into correlated agronomic traits to understand the effect of gender and location on farmers’ choice. We compare the predictive ability of grain yield (GY) with that of OA given by local farmers, finding that farmers’ OA can predict yield in untested environments with higher accuracy than agronomic measures. We then use forward genetics to map the genetic basis of OA, identifying QTL for phenology, yield, and farmer preference. Our results support the value of incorporating PVS in genomics-driven breeding to enhance genetic gain for local agriculture.

## Results and Discussion

### Agronomic Performance of Ethiopian Durum Wheat.

To test the added value of PVS in genomics-driven breeding, we focused on two sets of Ethiopian durum wheat genetic materials. A diversity panel (DP) of 400 varieties, mainly local landraces, was previously phenotyped for two consecutive seasons in two locations representative of Ethiopian agriculture (23) (*SI Appendix*, Fig. S1). PVS was conducted on DP lines involving men and women smallholder farmers with experience of wheat cultivation in the tested areas, collecting farmers’ OA on a Likert scale from one (poor) to five (very good). PVS data analysis on the DP was previously reported (15). Subsequently, landraces selected from the DP were intercrossed with a modern variety with international pedigree to produce a nested association mapping population, named the Ethiopian NAM (EtNAM) (11). Here, we focus on 1,200 recombinant inbred lines (RILs) belonging to 12 EtNAM families that were grown and phenotyped in three locations in Ethiopia in a fully replicated design (*SI Appendix*, Fig. S1). EtNAM RILs can be considered prebreeding materials in an early phase of varietal development. Both the DP and the EtNAM were phenotyped for yield, phenology, and yield components. PVS was conducted on the EtNAM on the same fields used for phenotyping, following the same procedure employed on the DP. Different farmer groups evaluated the genetic materials in each location. In all locations, the farmers involved in PVS were chosen to be representative of those residing in the area and to be expert wheat growers. Men and women were kept separated during PVS to untangle gender-specific differences in evaluating genetic materials. The DP and the EtNAM were both genotyped with dense molecular markers (11, 23).

To better frame the wheat genotypes’ performance in different locations, we conducted a climatological characterization of the experimental sites. Geregera (experimental site for both DP and EtNAM) and Kulumsa (experimental site for EtNAM) are in tepid submoist mid highlands. Hagreselam (experimental site for DP) is in the warm submoist lowlands, while Adet (experimental site for EtNAM) is in tepid moist mid highlands. The three EtNAM locations had different planting and harvesting dates and experienced different temperature and rainfall regimes throughout the cropping season. Kulumsa experienced the highest temperatures and the most consistent rainfall between flowering and full maturity of the EtNAM lines (*SI Appendix*, Fig. S2). Throughout the season, this site experienced the highest variability both in terms of weekly temperature range and in mean temperature (*SI Appendix*, Fig. S3). The experimental locations were chosen at sites commonly used by local breeding programs to test prebreeding materials and, regardless of local specificities, are all representative of the average climate of the wheat cropping area in the country (*SI Appendix*, Fig. S4).

### Farmers’ Preference is a Quantitative Trait.

**Fig. 1.**
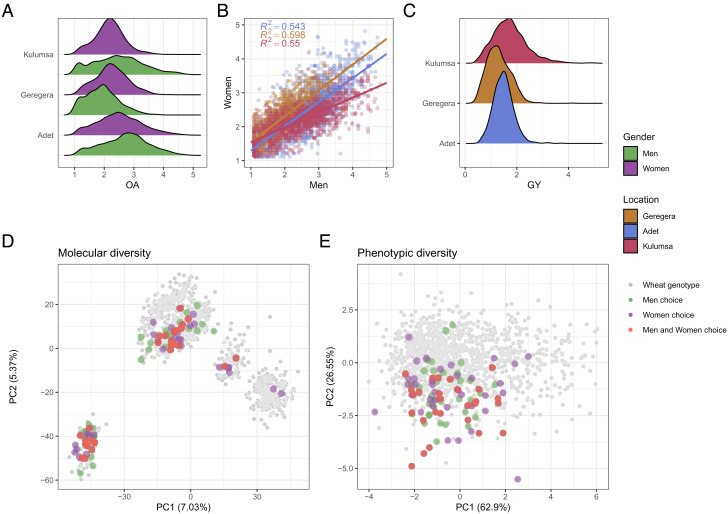
Diversity and agronomic performance in the EtNAM by BLUP value distributions. (*A*) Distribution of OA scores by gender and by location, with colors according to legend. (*B*) Distribution of men scores (x-axis) and women scores (y-axis), by location. A regression line is fitted to the score distribution for each location with colors according to legend. The model R^2^ is reported on top left with colors matching the distributions. (*C*) Distribution of GY performance by location. (*D*) Farmers’ top choice of genetic materials in the multivariate space of genetic diversity and (*E*) phenotypic diversity. Individual genotypes are marked in gray, while genotypes scoring above the 95th percentile of OA distribution according to men and women are highlighted in colors according to legend.

OA evaluations given by men and women farmers at each location showed similar distributions (Fig. 1*A*). Scoring was prudent in all the testing sites, with most materials ranking below average, so that “very good” EtNAM RILs were rare (*SI Appendix*, Fig. S5). The distribution of farmer evaluations was consistent across genders, though men provided higher OA than women in Adet and Kulumsa, and lower in Geregera (*P* < 0.001) (Fig. 1*B*). Although EtNAM materials achieved the highest yields at the Kulumsa site (Fig. 1*C*), farmers in Adet provided the most positive evaluations. Local communities provided scoring according to their own perception, and yield was not the sole component being considered while assigning OA values to genotypes. On the EtNAM, broad-sense heritability (*H^2^*) of farmer scores combined across genders (*H^2^* = 0.45) was comparable to that of yield (*H^2^* = 0.49) and of yield component traits (*SI Appendix*, Fig. S6), meaning that farmer preference for genetic materials is a repeatable, genetically determined trait that can be targeted by breeding programs and contribute to genetic gain. While evaluating OA in the EtNAM lines, men farmers provided higher heritability (*H^2^* = 0.54) than women (*H^2^* = 0.32, Table 1), as if their evaluation was less influenced by nongenetic factors. In sub-Saharan smallholder farming systems, men farmers mostly focus on agronomic traits, while women are more concerned with postharvest traits (24, 25). A different perception of OA by men and women is likely to affect the heritability of the evaluations, that are given on-field nearing flowering time. Regardless of their differences, men and women farmers selected a matching set of entries as their top choice, pointing to similar combinations of genetic and phenotypic trait values (Fig. 1 *D* and *E*). Among the genotypes scoring in the top 5% for OA, 26 were chosen by both men and women, while the remaining were chosen by either of the two groups (*SI Appendix*, Table S1). The genetic makeup of lines selected by both men and women farmers can be reconducted to the crossing that was used to develop the EtNAM (11). The EtNAM families most represented in the top farmer choice were N51, from the intercross of *Asassa* with the Italian variety *Bidi*, and N1 and N8, both from the intercross of *Asassa* with Ethiopian landraces.

**Table 1.** Broad-sense heritability (*H^2^*) of EtNAM traits measured across the tested locations. OA, overall appreciation; BM, biomass; DB, days to booting; DH, days to heading; DF, days to flowering; DM, days to maturity; GY, grain yield; NSPKPS, number of spikelets per spike; NTPP, total number of tillers; PH, plant height; SPL, spike length; SPS, seeds per spike; TGW, thousand grain weight.

Trait	Gender	*H^2^*
OA	Women	0.32
	Men	0.54
	Combined	0.45
BM	–	0.09
DB	–	0.62
DH	–	0.60
DF	–	0.59
DM	–	0.07
GY	–	0.49
NSPKPS	–	0.28
NTPP	–	0.05
PH	–	0.44
SPL	–	0.23
SPS	–	0.30
TGW	–	0.67

Farmers involved in the PVS were local wheat growers representative of the socioeconomic context in the surroundings of the experimental sites. Although they cannot be considered representative of the diversity of Ethiopian farming systems, they are representative of smallholder farmers living in the wheat-growing areas in the country (*SI Appendix*, Fig. S4). PVS studies conducted in Ethiopia on teff (26) and durum wheat (15) showed that farmers living in different agroecologies may provide matching evaluations of crop performance and preference. Lager panels of farmers involved with decentralized approaches also express similar patterns of varietal preference across locations, although with local specificities (21, 22). The consistency of evaluations given across farmer groups is rooted in their perception of crop performance in local farming conditions. Farmer evaluations were indeed correlated with agronomic traits in all locations (*SI Appendix*, Fig. S7). We found that OA was always positively correlated with yield and yield components, and mostly negatively correlated with phenology, suggesting that farmers preferred high yielding, tall and thick plants with early maturity, in agreement with previous assessments (19). The correlation between OA and GY was significant in all location and farmer groups and ranged from 0.41 to 0.66 (*SI Appendix*, Table S2). Yield and farmer appreciation were higher for modern varieties and EtNAM RILs than for landraces in all locations, though the advantage over landraces was less evident in Geregera, the most limiting environment. Some EtNAM RILs greatly outperformed both landraces and modern varieties for both yield and OA, supporting the breeding relevance of these genetic materials (*SI Appendix*, Fig. S8). Farmers’ OA was positively correlated with biomass and days to maturity in Kulumsa, but not in Adet and Geregera. The highest correlation was observed in modern varieties, followed by EtNAM RILs (*SI Appendix*, Fig. S9). Landraces grown in Geregera achieved yield and OA comparable to those of modern varieties, a hallmark of local adaptation. Modern wheat varieties that have a longer time to maturity, when grown in locations that allow a longer growing season like Kulumsa (*SI Appendix*, Fig. S2), express higher yield and are preferred by farmers. The relation between farmers’ preference and phenology is opposite in Geregera, a growing environment exposed to terminal drought. The selection of farmers thus depends on local adaptation and on the combination of traits in the materials of choice.

Farmers’ appreciation derives from a combination of traits depending on local uses and cropping conditions; thus, GY alone cannot summarize OA (15, 27). To test whether farmer evaluations could capture wheat stability across environments, we correlated OA values given in each location with yield stability indexes across locations (*SI Appendix*, Fig. S10). OA values given by farmers in Geregera, the most limiting environment, were significantly anticorrelated with yield variation across environments (*R^2^* = −0.29) and significantly correlated with measures of increased stability across locations. The same does not hold true for OA given by farmers in high potential areas (Adet, Kulumsa) (*SI Appendix*, Fig. S10), suggesting that OA expressed by wheat growers in challenging environments may capture not only yield (*R^2^* = 0.45 for men and *R^2^* = 0.47 for women), but also its stability across environments.

### Characterization of Farmers’ Choice for Breeding Applications.

We used a Plackett–Luce model (28–30) to bring farmer choice patterns into a ranking framework and evaluate their potential contribution to breeding decisions on the EtNAM. Building upon the results of our exploratory analyses, we considered two factors that could drive farmers’ choice. The first were farmers’ individual differences, such as different gender and locations influencing how farmers appreciate the different genotypes. The second factor were the individual characteristics of the wheat genotypes evaluated, as represented by best linear unbiased predictors (BLUPs) of measured traits. This approach to the characterization of farmer choice criteria could be at the core of a breeding program integrating PVS and is focused on identifying the relative worth of tested genotypes as a function of local performance and preference by farmers.

**Fig. 2.**
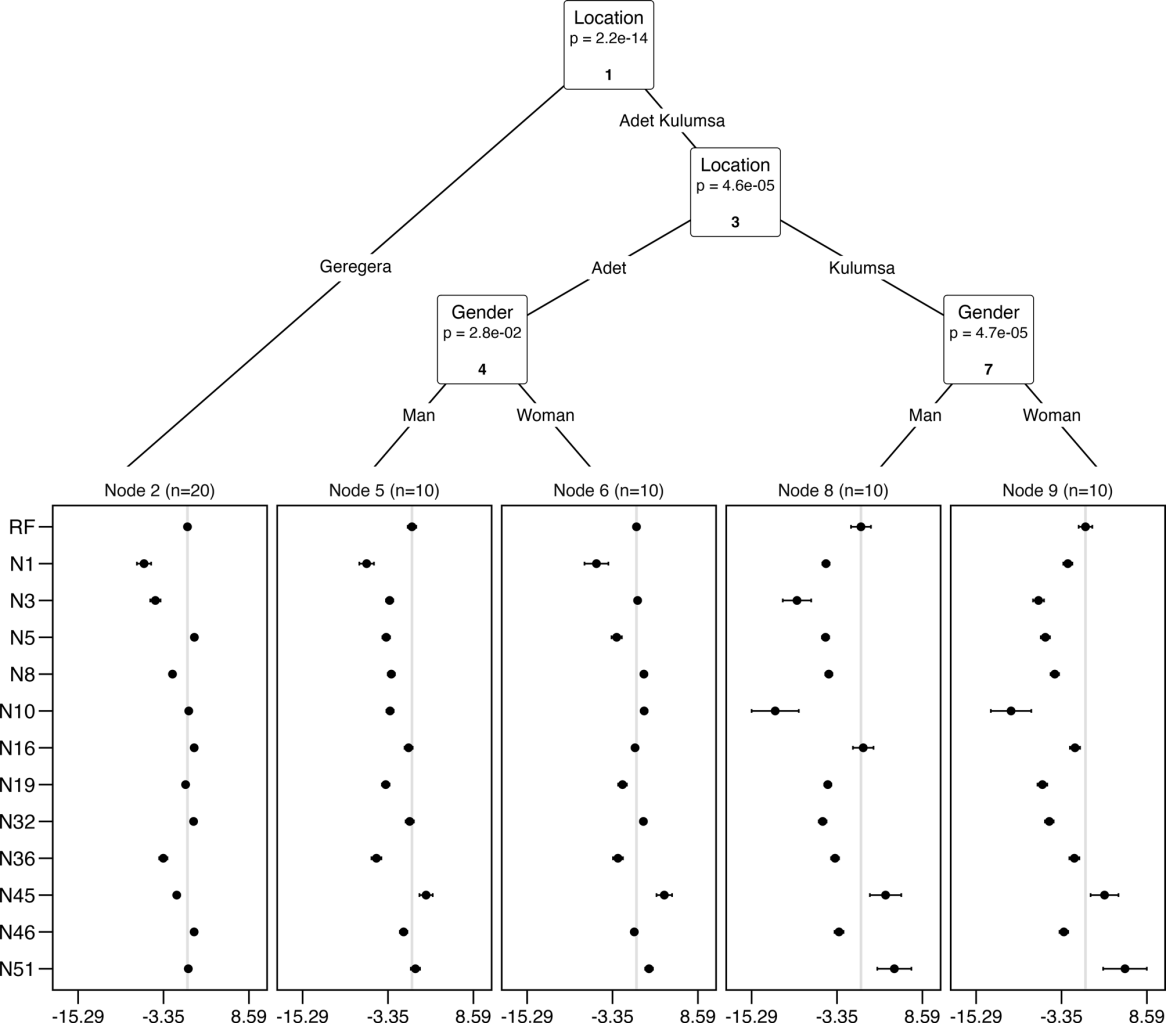
Breakdown of farmer choices on EtNAM genotypes. The x-axis reports the log-worth, the probability that each genotype within EtNAM families (y-axis) to be selected against the other genotypes. EtNAM families are reported with the corresponding code, N followed by a number. The entry RF represents the recurrent founder used to develop the EtNAM, the modern variety *Asassa*. The worth of RF was set at 0 for reference. Different groups classified by location and gender according to the model represent different choices in selecting genotypes. Drivers of farmers’ choices, based on agronomic metrics, are presented in *SI Appendix*, Table S1. Intervals are based on quasi-variance estimates. Data analysis was conducted on BLUP values.

Indeed, the worth of EtNAM prebreeding lines varied across RIL families, locations, and farmer groups (Fig. 2). Farmers in Geregera had a strong preference for genotypes with early maturity (days to booting and flowering), an indication that higher GY was not the priority for this group (*P* < 0.001) (*SI Appendix*, Table S3). In the other two locations, farmers’ choices were also influenced by gender. In Adet, women and men favored genotypes based on plant height (PH) and spike length (shorter spikes), while men also preferred genotypes with early maturity (flowering) but late booting. In Kulumsa, both men and women directed their choices toward genotypes with higher GY and biomass, and shorter days to flowering (DF) and heading (*P* < 0.001). Men in this location also preferred genotypes with shorter spikes, a trait valued in durum wheat breeding and associated to larger grains. In all cases, individual EtNAM RIL families could outperform the reference modern variety, *Asassa* (Fig. 2). Clearly, our study design based on quantitative scoring of appreciation with PVS cannot capture the subtle gender dynamics existing in local communities. These findings however can support the development of product profiles for demand-led breeding programs considering farmers’ drivers of variety selection as a mean to develop breeding materials with a higher likelihood for future adoption, fostering adaptation of cultivations to local uses and needs (31–34). In our case, EtNAM family N45 should be prioritized for Geregera and Adet, but not for Kulumsa. N51 had the highest worth in Geregera (Fig. 2). Further testing of these genetic materials may then be combined with on-farm decentralized data-driven approaches, which would allow the evaluation of genotypes in a broader set of environments in combination with socioeconomic drivers for selection and adoption (22). A similar design could allow further stratifying farmers’ features to inform more tailored and equitable varietal recommendations. Moreover, it could capture further details on socioeconomic and gender dynamics that influence variety selection at the household level, including gendered roles in agriculture and deeper inequalities related to access to agricultural innovation (34).

### GS Using Farmers’ Traditional Knowledge.

**Fig. 3.**
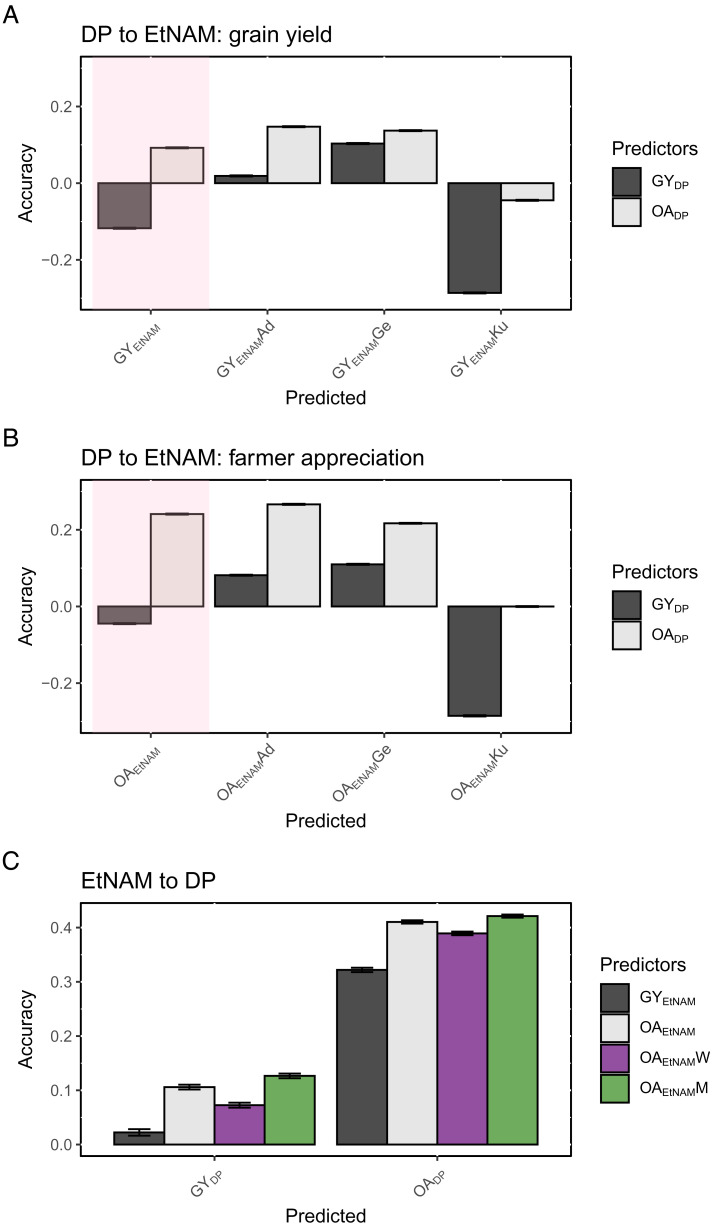
Accuracy of GS models considering GY and OA values measured in the DP and the EtNAM. (*A*) Prediction accuracy of a model trained on DP data and tested on GY in the EtNAM. The pink shading highlights combined data, while location-specific prediction accuracies are given separately. The accuracy of the prediction is reported on the y-axis with bars indicating SEM across 100 repetitions. The predictors are color coded according to legend, while predicted OA and GY measures are reported on the x-axis. (*B*) Prediction accuracy of a model trained on DP data and tested on OA in the EtNAM, plotted as in panel *A*. (*C*) Prediction accuracy of a model trained on the EtNAM and tested on DP data. OA values are split by gender (W, women; M, men) and combined across genders. Data analysis was conducted on BLUP values.

Having assessed that farmer scores were repeatable, heritable, and aligned with local performance of wheat genotypes, we tested whether PVS scores provided by men and women farmers could improve GS accuracy in the considered environments. To do this, we used GY and OA measured in the DP, that included genotypes that were used to produce the EtNAM (11), to predict the same phenotypes on the EtNAM. We found that a GS model trained on GY in the DP could not positively predict GY combined across locations in the EtNAM (Fig. 3*A*). However, the same GS model trained on OA in the DP positively predicted EtNAM GY with an accuracy of 0.09 (*SI Appendix*, Table S4). Although the magnitude and direction of prediction accuracy depended on the experimental sites, OA consistently outperformed GY in predicting EtNAM GY (Fig. 3*A*). Likewise, models trained on OA measured in the DP consistently outperformed GY in predicting farmers’ OA of EtNAM lines (Fig. 3*B*). In all cases, OA provided higher prediction accuracies, consistently above 0.20 on combined data and when predicting data measured in Adet and Geregera (*SI Appendix*, Table S4). Kulumsa, a CIMMYT test site representing high potential wheat-growing areas, was negatively predicted by the DP. This may be due to an opposite effect of allelic combinations that were suitable for highland cultivation, captured by the DP sites (Geregera, Hagreselam) and depending on local conditions. Despite the substantial inadequacy of DP data in predicting the EtNAM performance at Kulumsa, models trained on OA still performed better relative than GY when predicting yield and yield components. We found that OA scores could predict a combination of traits, with highest accuracies for biomass (0.15), SPS (0.15), PH (0.26), and thousand seed weight (0.27) (*SI Appendix*, Table S4). The prediction accuracy of both GY and OA improved when GS was restricted to the EtNAM germplasm, and the model was trained and tested across locations (*SI Appendix*, Table S5 and Fig. S11). These results suggest that OA may be especially relevant as a predictor when the training set and the test set of the GS are furthest apart, in line with farmers’ ability to capture wheat potential.

GS models could also work in the opposite direction. We found that EtNAM data could be used to predict DP performance, once again with higher accuracies achieved by models trained on OA and, in this case, particularly by OA evaluated by men (Fig. 3*C*). OA measured on the EtNAM positively predicted GY in the DP with an accuracy of 0.11 and could predict OA on the same panel with an accuracy of 0.41 (*SI Appendix*, Table S6). Conversely, GS models trained on GY measured in the EtNAM could predict GY in the DP only with an accuracy of 0.02. It is worth stressing that farmer groups evaluating the EtNAM and the DP were different, as different were farmer groups conducting the PVS experiment in each of the locations. Differences in GS prediction performance by gender reflected differences in the heritability of OA traits (Table 1) and may derive from different degrees of agreement within farmer groups.

PVS traits for farmer appreciation of earliness, spike morphology, and tillering capacity on the DP were collected with a method similar to that used for OA (20). PVS traits collected on the DP were correlated with components of agronomic performance: farmers preferred high yielding and early genotypes, with bigger seed size and thicker spikes (*SI Appendix*, Fig. S12). When PVS traits collected on the DP were used to predict phenology and yield components on the EtNAM, we found that spike morphology appreciation provided prediction accuracies comparable to those of OA for yield component traits, including biomass, number of spikelets per spike, PH, and thousand seed weight (*SI Appendix*, Fig. S13). The appreciation of earliness, which was strongly anticorrelated with DF and maturity, provided negative prediction accuracies for yield components and phenology.

When conducting PVS, farmers are simply inspecting plants in the field, nearing flowering time. The fact that OA predicts GY better than GY itself is striking and we could advance different hypotheses as to why this happens. A higher GS prediction accuracy may derive from the fact that farmer’s OA is given based on yield component traits with higher heritability than yield, thus achieving higher predictability. A higher accuracy may also derive from the fact that farmers provide OA based on their experience over multiple seasons, and thus capture the expected performance of genotypes in a similar environment with a greater accuracy than that can be derived from a limited number of GY observations. This is reinforced by the significant correlations that were observed between farmers’ OA and measures of yield stability (*SI Appendix*, Fig. S10). High prediction accuracies for OA derived from farmers’ ranking of wheat genotypes were also observed in a data-driven decentralized breeding approach (3D-breeding) focusing on local adaptation via on-farm testing (22). Our experimental design allows only a partial deconstruction of farmers’ appreciation on plant traits, but the consistency of the scoring system and accuracy achieved by the GS trained on OA suggest that PVS evaluations may capture underlying features of trait preference that are independent from farmer group, location, and gender. Further studies may expand the understanding of farmers’ decision-making processes (24, 35), and complement our findings to fully unravel the underlying reasons why farmers, at a glance, can successfully predict wheat performance across seasons and across location.

### Genetic Targets for Wheat Prebreeding.

**Fig. 4.**
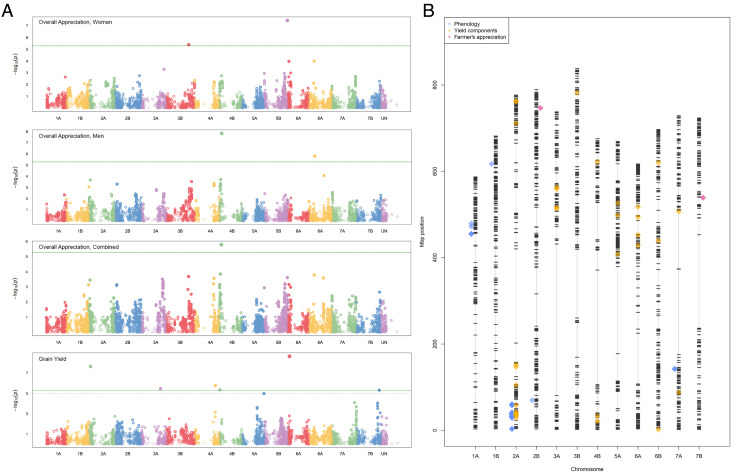
Genetic targets for participatory wheat improvement. (*A*) GWAS reporting marker trait associations for OA scored by women, OA scored by men, OA combined across genders, and GY. On the x-axis, SNP markers are arranged by their estimated physical position, with alternating colors corresponding to the 14 chromosomes of durum wheat plus an unmapped linkage group (UN). The y-axis reports the significance of the association, with SNPs surpassing the Bonferroni threshold (green line) marked as significant. The dashed green line, when present, represents a less stringent threshold for FDR-corrected *P*-values. (*B*) QTL mapping on individual EtNAM families. Markers included in EtNAM genetic maps are reported as black ticks according to their physical position. QTL are shown in colors according to legend and correspond to phenotypes grouped by phenology (DB, DH, DF, DM), yield components (GY, TGW, SPL, NSPKPS, SPS, PH, NTPP, BM), and farmers’ appreciation (OA). QTL markers are semi-transparent and have deeper shades of color proportionally to the number of EtNAM subfamilies in which they are detected. Data analysis was conducted on BLUP values combined across locations.

To further explore the potential contribution of PVS to molecular breeding, we used marker data developed on the EtNAM to conduct forward genetics approaches aimed at describing QTL for farmers’ preference and agronomic performance. The single-nucleotide polymorphisms (SNPs) used to genotype the EtNAM were assigned to their estimated physical positions mapping the array SNP probes to the *Triticum durum* reference genome (*SI Appendix*, Table S7). A genome-wide association study (GWAS) identified altogether 81 unique marker–trait associations (MTAs), 10 of which were for farmers’ OA, while the rest were for agronomic traits (*SI Appendix*, Table S8). Men and women OA scores identified significant associations on chromosomes 3B, 4B, 5B, and 6A. Gender differences in the evaluations were also reflected by the different set of loci targeted by men and women. The MTA for OA on chromosome 4B comapped with an MTA for GY (Fig. 4*A*). Previous studies reported a QTL hot spot for wheat yield stability on chromosome 4B (36), that could correspond to the MTA mapped by farmer scores. OA identified an MTA overlapping with GY also on the short arm of chromosome 5B (*SI Appendix*, Table S8), yet PVS MTAs were not exclusive to GY loci. OA comapped with thousand grain weight, a measure of seed size, in several loci including at approximately 650 Mb on chromosome 1B, 740 Mb on chromosome 3B (also comapping with days to maturity), and 689 Mb on chromosome 5B. OA matched phenology MTAs on the short arm of chromosomes 2A and 2B. Interestingly, an MTA for OA at approximately 175 Mb on chromosome 6B did not comap with any of the agronomic traits measured in this study. Farmers’ evaluation is based on a combination of different traits and may provide genetic targets beyond those for yield and yield components. Farmer scores could be thus used to complement molecular breeding to prioritize loci for breeding and support local adaptation and varietal acceptance.

To strengthen the identification of EtNAM subfamilies with higher relevance for prebreeding, we developed genetic maps specific for each of the original cross combination (*SI Appendix*, Table S9). We developed linkage groups in numbers ranging from 32 (EtNAM N16) to 46 (EtNAM N1), with a total length from 162 cM to 311 cM. Family N51, developed by the intercross of *Asassa* with the modern variety *Bidi*, is the only one lacking an Ethiopian landrace parent and showed the shortest genetic map span, with 1,187 markers included (x = 1,846, σ = 298.5). We used individual genetic maps to support QTL mapping specific to subfamilies, identifying 382 partially overlapping QTL (*SI Appendix*, Table S10). These individual genetic maps can be used to identify relevant haplotypes at QTL and locate significant associations on genetic backgrounds of interest to wheat breeding (Fig. 4*B*). In individual EtNAM families and in local experiments, OA was often mapped in association with phenology QTL, confirming the importance of early maturity traits for local farmers (*SI Appendix*, Fig. S14). These included chromosome 1A and 1B in EtNAM families N5, N19, N45, as well as several signals on chromosome 2A in families N16 and N5, which correspond to meta-QTL already reported in literature (37). On chromosome 1B, we identified OA QTL specific for the Adet environment in family N3. In this analysis, several OA QTL comapped with yield component traits including number of spikelets per spike, spike length, and GY (*SI Appendix*, Fig. S14). These positions may not be relevant to the trait in other genetic backgrounds (*SI Appendix*, Table S10) and may support the prioritization of EtNAM subfamilies for breeding. In both GWAS and QTL mapping on the EtNAM, accuracy is currently insufficient to support the identification of candidate genes. However, thanks to the rapid development of genomic tools on durum wheat (38) and associated species (39), further studies may explore QTL candidate genes and narrow down mapping intervals using a combination of forward and reverse genetics approaches (40, 41).

### Implications for Breeding Programs.

Data-driven methods are causing a transformational change in breeding. The availability of large-scale data including those deriving from genomics, phenomics, and remote sensing discloses new possibilities to accelerate genetic gain and deliver innovation tailored to end users (21). Our data show that, in a quantitative breeding framework, PVS data can add to the phenotypic characterization of tested materials to enhance selection accuracy for target environments. The involvement of smallholder farmers has the advantage of targeting local adaptation in challenging environments, prioritizing genetic materials with higher appreciation and therefore potential for uptake by local farmers. Farmer evaluations, however, cannot be the only driver of selection. There is the need for different data and approaches to come together into a coherent data-driven dimension bringing together farmers, breeders, and data scientists.

GS coupled with PVS could be used at early stages of the breeding pipeline. The resulting selection of lines, reinforced by quantitative data about farmers’ preference, can be moved on farm to test their performance in a larger set of environments closer to the intended use (42) while informing about farmers’ choice processes (15). GS could then be moved in a decentralized framework to improve selection accuracy and genetic gain via 3D-breeding (22). Indeed, the PVS approach described here still relies on a centralized breeding system that brings several limitations. These include high cost per datapoint and limited capacity for representation of environmental variation (43). Moreover, experimental stations are seldom representative of true cropping conditions in smallholder farmer fields, which typically make use of lower fertilizer rates and less intense weeding. This may skew the farmers’ evaluation to follow more closely yield potential and consequently bias the evaluation of materials. However, thanks to its substantial alignment with ongoing major centralized breeding programs, the combination of on-station PVS coupled with GS could be readily integrated with low attrition to conduct early selection of genetic materials to be then validated on farm (22). The involvement of much larger number of farmers in a decentralized evaluation scheme building upon PVS-driven GS would allow to gather a broader representation of end users and consumers and further contribute to the tailoring of seed innovation for smallholder farming systems. A formal integration of PVS in modern breeding at all levels may also reinforce farmer organizations (44), recognized as a major player in supporting food security in emerging countries exposed to climate change. Finally, by better characterizing the role of gender in varietal adoption, researchers could contribute to empowering local women and improving their access to agricultural innovation, a hallmark of food security (45).

## Conclusions

We found that OA measures derived from PVS can be used in genomics-assisted breeding, either being explicitly factored in GS models to improve prediction accuracy of yield, or by mapping genomic loci associated to farmers’ OA. We do not underestimate the need to reach a better understanding of farmers’ decision processes and preferences regarding local cultivation. The intercrossing of traditional genetic materials with an international breeding line in the EtNAM generated lines that both met farmer needs and provided increased production traits in target environments. Multiparental populations such as the EtNAM are at once research tools and prebreeding materials and represent an avenue to leverage local agrobiodiversity for crop improvement (46). Currently, the EtNAM RILs lines are being evaluated for prebreeding in different locations in Ethiopia.

In Ethiopia, durum wheat is being rapidly replaced by semi-dwarf bread wheat varieties introduced since the 1980s (47). Durum wheat is cultivated on a fraction of the wheat area in the country, more than 99% of it represented by farmer varieties is mainly used for traditional preparations despite lower productivity as compared to bread wheat (48). Ethiopian durum wheat is markedly separated from the international allele pool (23), and a GS coupled with PVS could help unlocking its breeding potential for national and international programs. Farmers’ acceptance of new varieties is crucial to determine breeding success (49), and we believe that GS methods based on participatory approaches may improve local adaptation of genotypes. This calls for an effort requiring multidisciplinary approaches, ranging from genomics to agronomy, to climate and social sciences (32). This experiment shows that the traditional knowledge of farmers may capture varietal potential for performance and adaptation, contributing to higher prediction accuracies, particularly in challenging environments.

## Materials and Methods

### Plant Materials.

Plant materials in this study derive from two sources: a DP of 400 Ethiopian wheat genetic materials and a nested association mapping population (EtNAM) originated from a selection of landraces from the DP. The DP was sourced from the ex situ wheat collection at the Ethiopian Biodiversity Institute (EBI) and is composed of 372 wheat landraces and 28 improved varieties derived from breeding. Landraces were for the most part durum wheat (*Triticum turgidum* subsp. *durum* L.) but some were bread wheat (*Triticum aestivum* L.) (23). Landrace accessions as obtained from the EBI collection were purified in open field during the 2011 main season by selecting a representative spike for each entry and reproducing it in the following seasons. Seedlings from five seeds from a single spike were germinated and used to extract genomic DNA with a GenElute Plant Genomic DNA Miniprep Kit (Sigma‐Aldrich). DNA extraction was conducted in Ethiopia at the laboratories of Mekelle University, Tigray. DNA was pooled among seedlings from the same accessions and genotyped with the Infinium 90k wheat chip (50) at TraitGenetics GmbH (Germany), with details given in the study by Mengistu et al. (23).

The EtNAM was developed intercrossing 50 Ethiopian landraces plus an Italian improved variety (*Bidi*) with the improved durum wheat variety *Asassa* with international background, all belonging to the DP. Selection of the EtNAM founder lines was aimed at maximizing i) genetic diversity, ii) segregation of traits of agronomic relevance, and iii) farmer preference of genetic materials. Details on the development of the population are given in the study by Kidane et al. (11). EtNAM RILs were derived from single seed descent until F_8_. A subset of 1,200 EtNAM RILs were selected from 12 families, 100 RILs each, to represent the broader diversity of the population. One of the selected 12 EtNAM families derives from the cross between *Asassa* and the Italian improved variety *Bidi* (family N51). The remaining families (N1, N3, N5, N8, N10, N16, N19, N32, N36, N45, and N46) were derived from *Asassa* and landrace parentals. EtNAM RILs in the selection were genotyped with a subset of 13,000 most informative markers from the Infinium 90k wheat chip (50) at TraitGenetics GmbH (Germany). Details on the selection of the EtNAM families and their genotyping are given in the study by Kidane et al. (11).

### Field Trials and Measurement of Agronomic Traits.

The DP and the EtNAM were evaluated in multiple locations using similar experimental designs. Details on the agronomic management of field trials are given in the *SI Appendix*. The DP was phenotyped in the main season in 2012 and 2013 in Geregera (11°40′N/38°52′E) and Hagreselam (13°38′N/39°10′E) (*SI Appendix*, Fig. S1). GY was measured as grams of grains produced per plot and then converted into t·ha^−1^. Full details are given in the study by Mengistu et al. (23). The EtNAM was phenotyped in the main season of 2016 in Adet (11°15′N/37°29′E) and in Geregera (11°40′N/38°52′E), and in the main season of 2017 in Kulumsa (8°01′N/39°09′E) (*SI Appendix*, Fig. S1). On the EtNAM, field technicians measured days to booting (DB), heading (DH), flowering (DF), and maturity (DM) when 50% of each plot reached such phenological stage. After harvesting, five plants per plot were selected at random and used to measure PH (cm), number of total tillers per plant (NTPP), spike length (SPL, cm), number of seeds per spike (SPS), and number of spikelets per spike (NSPKPS). Biomass (BM, t ha^−1^), (GY, t ha^−1^), and thousands grain weight (TGW, grams) were measured on full plots’ harvest. Methods for the climatic characterization of experimental locations are given in the *SI Appendix*.

### Participatory Variety Selection.

Participatory variety selection (PVS) was conducted when half of the plots reached flowering stage so as to maximize the discernment capacity between plots. Farmers taking part in the PVS were wheat growers living in the surroundings of each of the phenotyping locations. Before PVS, farmers were divided by gender and sorted in groups with five people each. Farmer groups were conducted across the field from random entry points and asked to evaluate their overall appreciation of each individual plot, defined as OA, on a Likert scale from 1 (poor) to 5 (very good). OA may be expressed as an answer to the following question: “how much do you like this plot?”. Therefore, OA scores are not bound to predefined traits, but rather reflect a measure of how much a farmer likes a specific variety. Farmer scores were individually recorded and numerically analyzed as phenotypes with details in the section below. Details about farmer selection and scoring procedure are given in the *SI Appendix*.

### Phenotypic Data Analysis and Breakdown of Farmers’ Preference Choice Processes.

All data analyses were conducted in R (51). Agronomic and PVS traits collected on the DP and on the EtNAM were used to derive best linear unbiased prediction (BLUP) values with R/ASReml (52). BLUP models and equations are given in the *SI Appendix*. Pearson’s correlations were performed among BLUPs for PVS and agronomic traits on the DP and the EtNAM if not stated otherwise. To avoid incurring in the Simpson’s paradox—that is, misleading correlation estimates due to stratification in the data—we independently performed correlation according to groupings in the data (e.g., gender, location, and year). Stability metrics of EtNAM genotypes performance across different locations were computed following the multitrait stability index method (53) implemented in R/metan (54). The rationale of this analysis was to see whether farmer scoring measured in each location would correlate with yield stability that is computed on agronomic traits across locations. This would be a sign that farmers may capture adaptation potential of genotypes and thus performance across environments. Yield stability was measured according to Shukla’s (55) and Annicchiarico’s (56) methods. The Multi-Trait Stability Index was also used (53). Measures of yield variation as coefficient of variation and genotypic variance were also included. Farmers’ OA measures given in each location were then used in a Spearman’s correlation with stability indexes.

We used a Plackett–Luce model (28–30) to estimate farmers’ appreciation on genotypes. The model applies Luce’s Axiom that estimates the probability that a given genotype has in outperforming all the other genotypes in a set. This probability may be interpreted as the worth of any given genotype. Farmers’ assessments were converted to pairwise comparisons, in which genotypes with a higher value (from 1 to 5) got a “win” when compared to another genotype with a lower value. To optimize model convergence, we did not consider ties in the pairwise comparisons. Data from each individual farmer were then aggregated by RIL families. We considered two main effects that could potentially drive farmers’ choice. The first were farmers’ individual differences, such as different gender and locations influencing how farmers appreciate the genotypes. The second factor were the individual characteristics of genotypes, reported by BLUPs (57) Details on the procedure are given in the *SI Appendix*.

### GS Model.

Allele calls were filtered for failure rate (<20%) and heterozygosity (<50%) in both DP and EtNAM data. R/rrBLUP (57) was used to conduct GS with the GBLUP model. SNPs were imputed with the mean method in the *A.mat*() function in rrBLUP. The set of SNPs overlapping among the DP and EtNAM was used to run the GS. The selection model was run according to different scenarios. To test whether farmers’ knowledge could accurately predict wheat performance, we focused our GS on OA and GY. The DP was used to predict EtNAM performance by training the model on 100 random subsets of 4/5 of the DP data. All traits measured on the DP were used as predictors over EtNAM traits. In a second scenario, a cross validation was performed across locations predicting the EtNAM over EtNAM data, iteratively using data collected in one of the locations to predict performances in the remaining two locations. In a third scenario, EtNAM data were used to predict DP data by training the model on 100 random subsets of 4/5 of the EtNAM data, using all partitions of computed BLUPs. Finally, an extended set of PVS evaluations collected on the DP was used to predict phenology and yield component traits on the EtNAM as in the first scenario described above. In all prediction scenarios, mean prediction accuracy and SE were derived from Pearson’s correlations between individual predictions and observed data.

### Forward Genetics and Genetic Map Construction.

The same SNP set used in GS was employed in a GWAS focusing on BLUPs for agronomic traits and farmer appreciation traits. Sequences of SNP marker probes were obtained by TraitGenetics GmbH (Germany) and mapped on the Svevo reference genome (38) available at the European Nucleotide Archive (Project: PRJEB22687) using bwa (58) and samtools (59) with no upstream filtering, obtaining a hypothetical genomic physical position for each marker. The GWAS was run in R/GAPIT (60), using the Bayesian-information and LD iteratively nested keyway (BLINK) method (61). The first three principal components of SNP data were used as covariates. The QQ plots produced by the model were manually inspected to evaluate model fit. A Bonferroni multiple test threshold was used to determine significance at a nominal *P*-value of 0.05. A less stringent FDR-corrected p-value threshold (62) is reported on plots when relevant.

SNPs were loaded in JoinMap® 5 (63) to construct linkage maps. Genotype data were filtered, allowing a marker segregation distortion (departure from the expected 1:1 segregation ratio, considering a:b genotypes) up to a threshold P = 1E−05, corresponding to a χ^2^ value of >23.9. For each family, linkage groups (LGs) were determined using the group function including markers with a recombination frequency <0.35 and a minimum LOD = 6.0. LGs were selected to possibly include makers belonging to the same chromosome or markers from different chromosomes that were not separated at LOD 16. Details for marker cleaning and LG consolidation are given in *SI Appendix*.

Mapping of QTL was performed in R/qtl2 (64). Linkage maps were associated to SNP data and phenotypes for each EtNAM family. Pseudomarkers were included with step 1, and mapping was conducted with kinship correction estimated with the leave-one-chromosome-out method, i.e., on all chromones except the one on which mapping is performed. QTL were mapped with a linear mixed model with a polygenic effect estimated under the null hypothesis of no QTL. The 90th percentile of the permuted LOD score distributions with 1,000 permutations was used to define significant QTL. QTL confidence intervals were defined with a peak drop of LOD=1 and Bayes credible intervals at 0.9.

## Supplementary Material

Appendix 01 (PDF)

Dataset S01 (XLSX)

## Data Availability

Data management relied on R/tidyverse (65) and R/rgdal (66). Plotting made use of R/ggplot2 (67), R/raster (68), and R/patchwork (69). All scripts are available on the GitHub page of the corresponding author at https://github.com/mdellh2o/EtNAM.GS. Raw data are available through Dryad at https://doi.org/10.5061/dryad.w6m905qrv.
