# Supplementary material for: Genomics-driven breeding for local adaptation of durum wheat is enhanced by farmers’ traditional knowledge
Source: Proc Natl Acad Sci U S A. 2023 Mar 27;120(14):e2205774119. doi: 10.1073/pnas.2205774119 (PMC10083613; doi:10.1073/pnas.2205774119)
Supplement: Supplementary file 1 — Appendix 01 (PDF) [file pnas.2205774119.sapp.pdf]

Supplementary Materials for:

Genomics-driven breeding for local adaptation of durum wheat is enhanced by farmers' traditional knowledge

Cherinet Alem Gesesse, Bogale Nigir, Kauê de Sousa, Luca Gianfranceschi, Guido Roberto Gallo, Jesse Poland, Yosef Gebrehawariat Kidane, Ermias Abate Desta, Carlo Fadda, Mario Enrico Pè, Matteo Dell'Acqua*

Correspondence to: m.dellacqua@santannapisa.it

This file includes:

Supplementary Methods

Supplementary Figures from Fig. S1 to Fig. S14

Supplementary Methods

Agronomic management of field trials

The DP trial was laid out in an alpha lattice design with 400 replicated entries (800 plots in total). Field management was conducted as per local guidelines and weeding was conducted manually. Plots were made of four rows 2.5 m long, with a seeding rate of 100 kg·ha^{-1}. 100 kg·ha^{-1} diammonium phosphate and 50 kg·ha^{-1}urea were applied at sowing, and additional 50 kg·ha^{-1} of urea were provided at tillering.

1,200 EtNAM RILs and 25 checks were laid out in a replicated alpha lattice design in each location (2,450 plots in total). Checks included modern varieties, local checks, and breeding materials. In Adet, plots were made of three rows 2 m long. In Geregera, plots were made of four rows of 1.5 m long. In Kulumsa, plots were made of four rows of 2 m long. Seeding rate was 100 kg·ha^{-1}. Agronomic management practices were applied following the same guidelines in all experimental units in every location. The fertilizer rate was of 92 N + 46 P_2O_5 kg·ha^{-1}. Half-dose urea was applied at planting and half-dose was applied at tillering, for a total rate of 150 kg·ha^{-1}. NPS was used at a rate of 121 kg ha^{-1}.

Climate similarity between stations and the target environment

The experimental locations were characterized for their climatic diversity with R/climatrends (1) and R/chirps (2), using data from NASA Power (3). Location-specific weather data was extracted considering sowing date and harvesting date specific for each site. For temperature, we considered minimum and maximum daily temperature (°C), averaged over weeks. For rainfall, we considered the simple

precipitation intensity index (SPII, mm/day), deriving from the sum of precipitation in wet days (days with >1mm of precipitation) over a week, and dividing that by the number of wet days in the same period. Climate similarity was computed with the analogue method proposed by Hallegatte et al. (4) to describe the *climatic analogy* between the EtNAM phenotyping stations and the general area for durum wheat cultivation across Ethiopia. We used temperature and precipitation data derived from WorldClim (5) to generate geographic extrapolation on climate similarity of the stations' climate during the growing season of durum wheat (June to January). Climate similarity was estimated for each station separately and then combined into a single map. A threshold of 0.2 (based on maximum specificity and maximum sensitivity) was used to eliminate areas with lower potential for durum wheat cultivation. The analysis was implemented using R/analogues (6).

<u>Participatory Variety Selection</u>

Farming communities in all EtNAM and DP phenotyping locations (Adet, Geregera, Hagreselam, and Kulumsa) are accustomed to interacting with regional research centers with participatory approaches. Those participating in the study were chosen among volunteers in the communities surrounding the fields, maintaining a balance of age, socioeconomic condition, and gender. Men and women were chosen from different households to avoid family bias. All were wheat growers with experience of continuous wheat cultivation. In each field, local research institutions provided rapporteurs speaking the local language to side the farmers in the evaluations. PVS in the DP involved a total of thirty farmers in each location (15 men and 15 women), while PVS in the EtNAM involved ten farmers in each location (5 men and 5 women). In the DP, farmers expressed other traits of appreciation, namely earliness (EA), tillering capacity (TL), and spike morphology (SM), answering a question in the form of “how much do you like the [trait] of this plot?”. To avoid scoring bias and cross-influence from power dynamics within groups, farmers were asked to express their scoring simultaneously. Further details may be found in Mancini et al. (16).

<u>Derivation of best linear unbiased prediction (BLUP) values</u>

We obtained best linear unbiased prediction (BLUP) values for metric traits using the model in Eq. S1:

$$y_{ijk} = \mu + g_i + s_j + l_k + gl_{ik} + gs_{ij} + sl_{jk} + e \qquad \text{Eq. (S1)}$$

where the observed phenotypic value is y_{ijk}, μ is the overall mean of the population, g_i is the random effect for the ith genotype *g*, s_j is the random effect for the jth season (*i.e.* year) *s*, l_k is the fixed effect for the kth location *l*, gl_{ik} and gs_{ij} are the random effect interactions between genotype and location and between genotype and year, sl_{jk} is the random interaction between year and location, and e is the error. For calculation of BLUPs with a single location and a single year, the data was sub-set by location/year

and the model in Eq. (S1) was simplified accordingly. Broad-sense heritability (H^2) of agronomic traits was derived from the variance component estimates deriving from Eq. (S1) as follows:

$$H^2 = \frac{\sigma_g}{\left(\sigma_g + \frac{\sigma_{gl}}{n_{loc}} + \frac{\sigma_{gs}}{n_{year}} + \frac{\sigma_e}{n_{rep} * n_{loc} * n_{year}}\right)} \qquad \text{Eq. (S2)}$$

In Eq. (2), σ_g is the variance component of genotypes, σ_{gl} is the genotype by location variance, σ_{gs} is the genotype by year variance, and σ_e is the error variance. n_{loc}, n_{year}, and $n_{rep}$are the number of locations, years and replications, respectively. For calculation of H^2 within years and locations, Eq. (S2) was simplified accordingly.

The derivation of PVS BLUPs and H^2 was similar to that used for agronomic traits except for the fact that gender and farmers performing the scoring were taken into account. PVS BLUPs were obtained from the model in Eq. (S3):

$$y_{ikmf} = \mu + g_i + l_k + p_m + d_f + gl_{ik} + gp_{im} + gd_{if} + pl_{mk} + glpd_{ikmf} + e \qquad \text{Eq. (S3)}$$

Where y_{ikmf} is the observed PVS score, and μ, g_i, l_k, and gl_{ij} are as in Eq. (S1). p_m is the random effect for the mth gender and d_f is the random effect for the fth farmer providing the scoring. Accordingly, gp_{im} and gd_{if} are the random effect of the interaction between genotype and gender and genotype and farmer, respectively. pl_{mk} is the random interaction between gender *m* and the kth location, and $glpd_{ikmf}$ is the random interaction between genotype, gender, location, and the famer providing the scoring. For calculation of BLUPs specific for gender, location and gender by locations, Eq. (S3) was simplified accordingly. H^2 for PVS traits was derived from the following formula:

$$H^2 = \frac{\sigma_g}{\left(\sigma_g + \frac{\sigma_{gl}}{n_{loc}} + \frac{\sigma_{gm}}{n_{gender}} + \frac{\sigma_{gf}}{n_{farmer}} + \frac{\sigma_e}{n_{rep} * n_{loc} * n_{gender} * n_{farmer}}\right)} \qquad \text{Eq. (S4)}$$

In Eq. (S4), σ_g is the variance component of genotypes, σ_{gl} is the genotype by location variance, σ_{gm} is the genotype by gender variance, σ_{gf} is the genotype by farmer variance, and σ_e is the error variance. n_{loc}, n_{gender}, n_{farmer}, and n_{rep} are the number of locations, genders, farmers, and replications, respectively. For calculation of H^2 by gender and by location, Eq. (4) was simplified accordingly.

Rank-based assessment of farmers' overall appreciation

Farmer overall appreciation of genotypes was assessed using the Plackett-Luce model, independently proposed by Luce (7) and Plackett (8). The model applies the Luce's Axiom that estimates the probability that, in our context, a given genotype has in outperforming all the other genotypes in a set. The Plackett-Luce is implemented in R/PlackettLuce (9). We report log-worth, the probability of outperforming a

reference genotype, as a raw output from Plackett-Luce model. We define this probability the *worth* of a genotype. To account for the effect of gender and location on genotype preference, we applied a model-based recursive partitioning approach (10). To account for the effect of genotype features on farmers' evaluation, we applied the Alternating Directions Method of Multipliers (ADMM) algorithm (11), which estimates the linear predictor for log-worth by genotype covariates. This approach helps in explaining the intrinsic characteristics of the genotypes influencing the selection of genotypes by farmers. We used BLUPs derived from the biomass, days to booting, days to flowering, days to heading, grain yield, plant heigh, spike length, seeds per spike and thousand seed weight as genotype covariates in the ADMM analysis.

<u>Genetic map development and filtering</u>

Marker data on the EtNAM was divided by family and converted in A, B, H notation with the ABH plugin in Tassel (12), with A being the recurrent founder *Asassa*. Prior map construction, each marker was renamed including the chromosome name and a sequential number (*e.g.* c.1B.m.0063) according to the physical mapping on the Svevo sequence (13) to easily verify the correspondence between genetic and physical maps. Markers were input to linkage group (LG) construction using the fixed order deriving from physical positions, removing markers mapping on different chromosomes. Multipoint maximum likelihood algorithm and Haldane mapping function were used. Each LG map was then manually checked for suspicious marker scores through the inspection of the graphical genotypes and comparing the maps with the previously available durum wheat consensus map (14). Markers whose *nearest neighbour stress* score was higher than ±2.0 were individually examined and eliminated when in contrast with the adjacent markers. Mapping was repeated until the stress score was less than ±2.0 for all markers or scores >±2.0 were due to the presence of large genetic distances between flanking markers. LG groups were ordered along chromosomes according to the average estimated physical position of the markers in the LG.

Supplementary Methods References

1. K. de Sousa, J. van Etten, S. Ø. Solberg, climatrends: Climate Variability Indices for Ecological Modelling (2020).
2. K. de Sousa, A. H. Sparks, W. Ashmall, J. van Etten, S. Ø. Solberg, chirps: API Client for the CHIRPS Precipitation Data in R. *The Journal of Open Source Software* **5**, 2419 (2020).
3. A. H. Sparks, nasapower: A NASA POWER Global Meteorology, Surface Solar Energy and Climatology Data Client for R. *The Journal of Open Source Software* **3**, 1035 (2018).

4. S. Hallegatte, *et al.*, Using climate analogues for assessing climate change economic impacts in urban areas. *Climatic Change 2007 82:1* **82**, 47–60 (2007).

5. S. E. Fick, R. J. Hijmans, WorldClim 2: new 1-km spatial resolution climate surfaces for global land areas. *International Journal of Climatology* **37**, 4302–4315 (2017).

6. D. Lodono, E. Jones, J. Ramirez-Villegas, analogues: Identification of Climate Analogues (2020).

7. V. Cane, R. D. Luce, Individual Choice Behavior: A Theoretical Analysis. *J R Stat Soc Ser A* **123**, 486 (1960).

8. R. L. Plackett, Analysis of Permutations. *J Appl Stat* **24**, 193–202 (1975).

9. H. L. Turner, J. van Etten, D. Firth, I. Kosmidis, Modelling rankings in R: the PlackettLuce package. *Comput Stat* **35**, 1027–1057 (2020).

10. A. Zeileis, T. Hothorn, K. Hornik, Model-based recursive partitioning. *Journal of Computational and Graphical Statistics* **17**, 492–514 (2008).

11. İ. Yıldız, *et al.*, Fast and Accurate Ranking Regression. 77–88 (2020).

12. P. J. Bradbury, *et al.*, TASSEL: Software for association mapping of complex traits in diverse samples. *Bioinformatics* **23**, 2633–2635 (2007).

13. M. Maccaferri, *et al.*, Durum wheat genome highlights past domestication signatures and future improvement targets. *Nat Genet* **51**, 885–895 (2019).

14. M. Maccaferri, *et al.*, A consensus framework map of durum wheat (Triticum durum Desf.) suitable for linkage disequilibrium analysis and genome-wide association mapping. *BMC Genomics 2014 15:1* **15**, 1–21 (2014).

Supplementary Figures

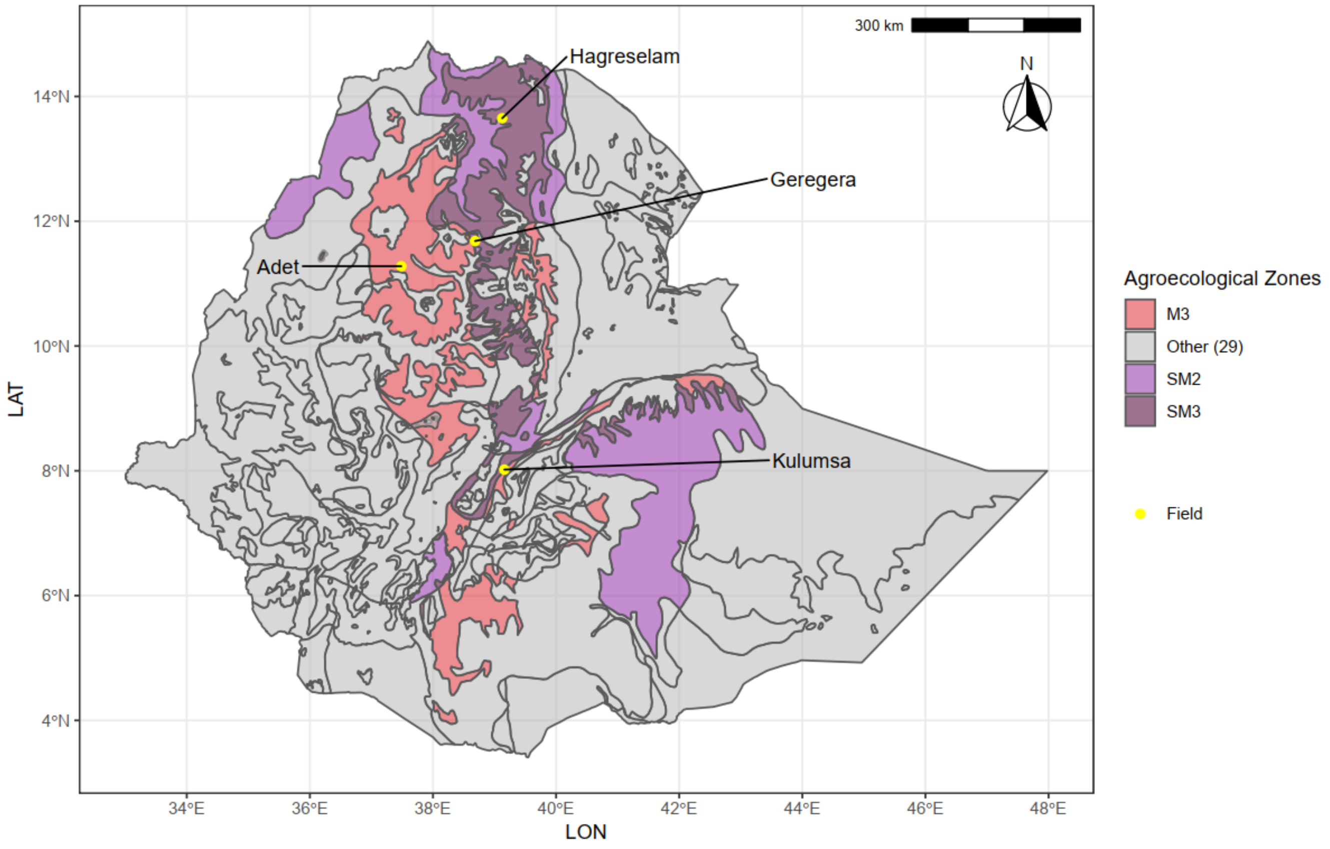

Fig. S1. Location of the phenotyping fields and agroecological zonation of Ethiopia. The agroecological zones in which phenotyping locations are located are colored according to legend; the remaining ones are depicted in gray. The diversity panel (DP) was evaluated in Geregera and Hagreselam. The EtNAM was evaluated in Geregera, Adet, and Kulumsa. M3, Tepid moist mid highlands (Adet); SM2, Warm sub-moist lowlands (Hagreselam); SM3, Tepid sub-moist mid highlands (Geregera, Kulumsa).

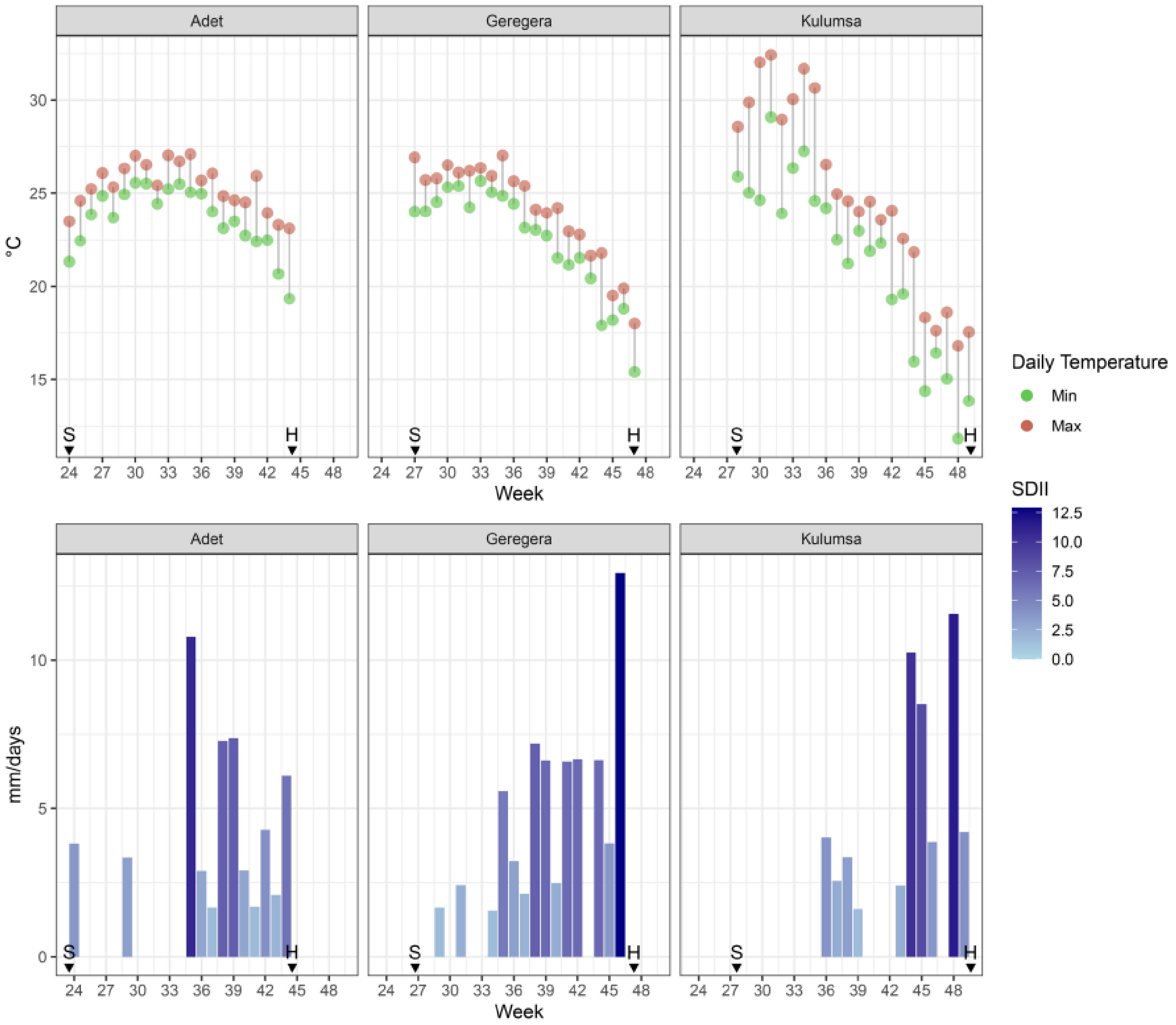

Fig. S2. Temperature and rainfall regimes at the EtNAM phenotyping locations during the cropping seasons object of this experiment. The top graphs show location-specific maximum and minimum weekly temperatures in the cropping season. The three locations are shown in separate panels, each reporting the sowing week (S) and harvest week (H). As compared to Adet and Geregera, Kulumsa experienced a unique temperature regime, higher at the beginning of the season and lower close to harvesting. The bottom row reports the simple precipitation intensity index (SDII), as millimeters (mm) of rain per day, in the three locations, with bars colored according to SDII values. The SDII is calculated on a week basis by summing the precipitation in wet days (days with >1mm of precipitation) and dividing that by the number of wet days in the period.

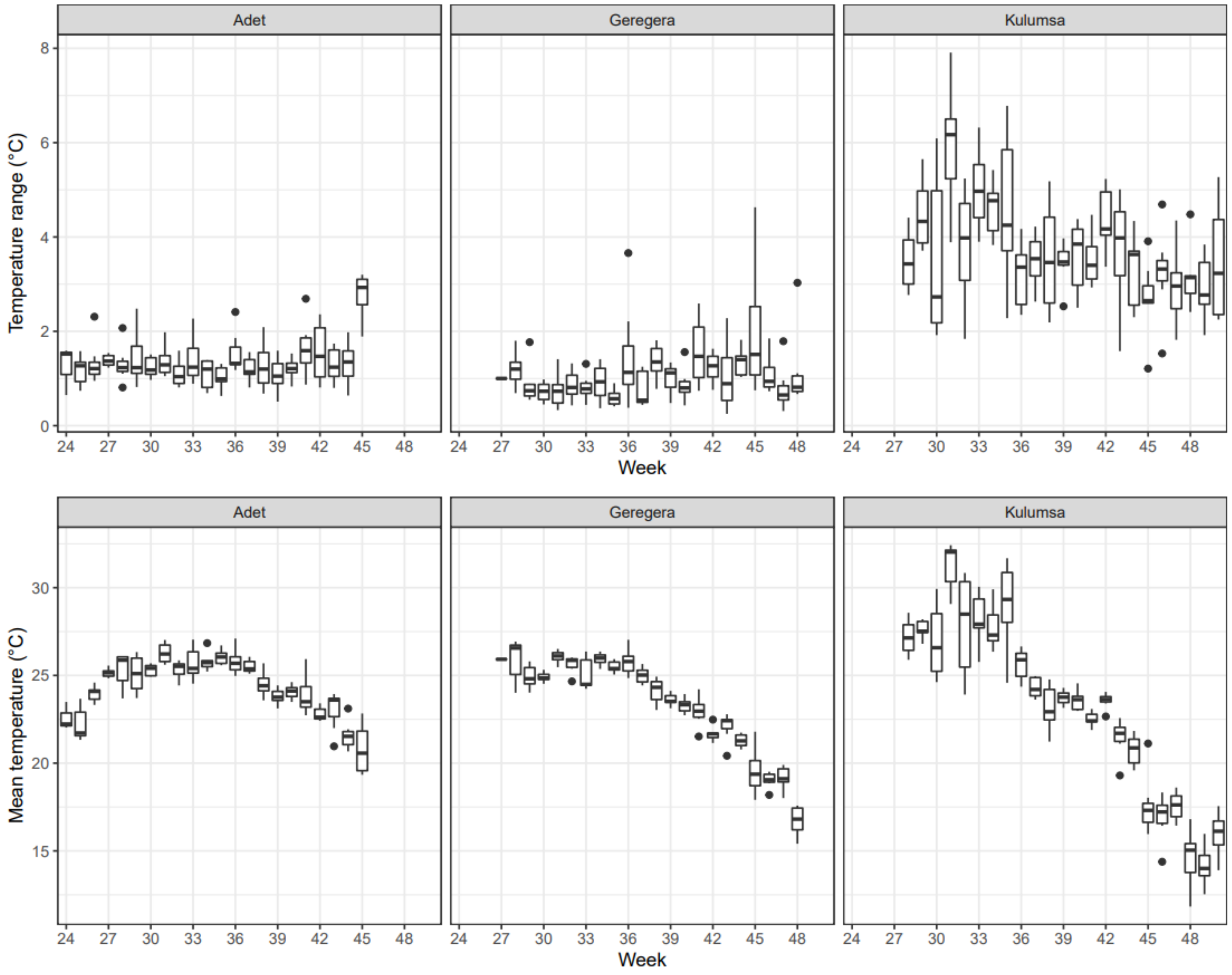

Fig. S3. Weekly temperature range (top panels) and mean temperature (bottom panels) at the EtNAM phenotyping locations during the cropping seasons object of this experiment. The three locations are shown in separate panels, each boxplot represent the distribution of the values within each week.

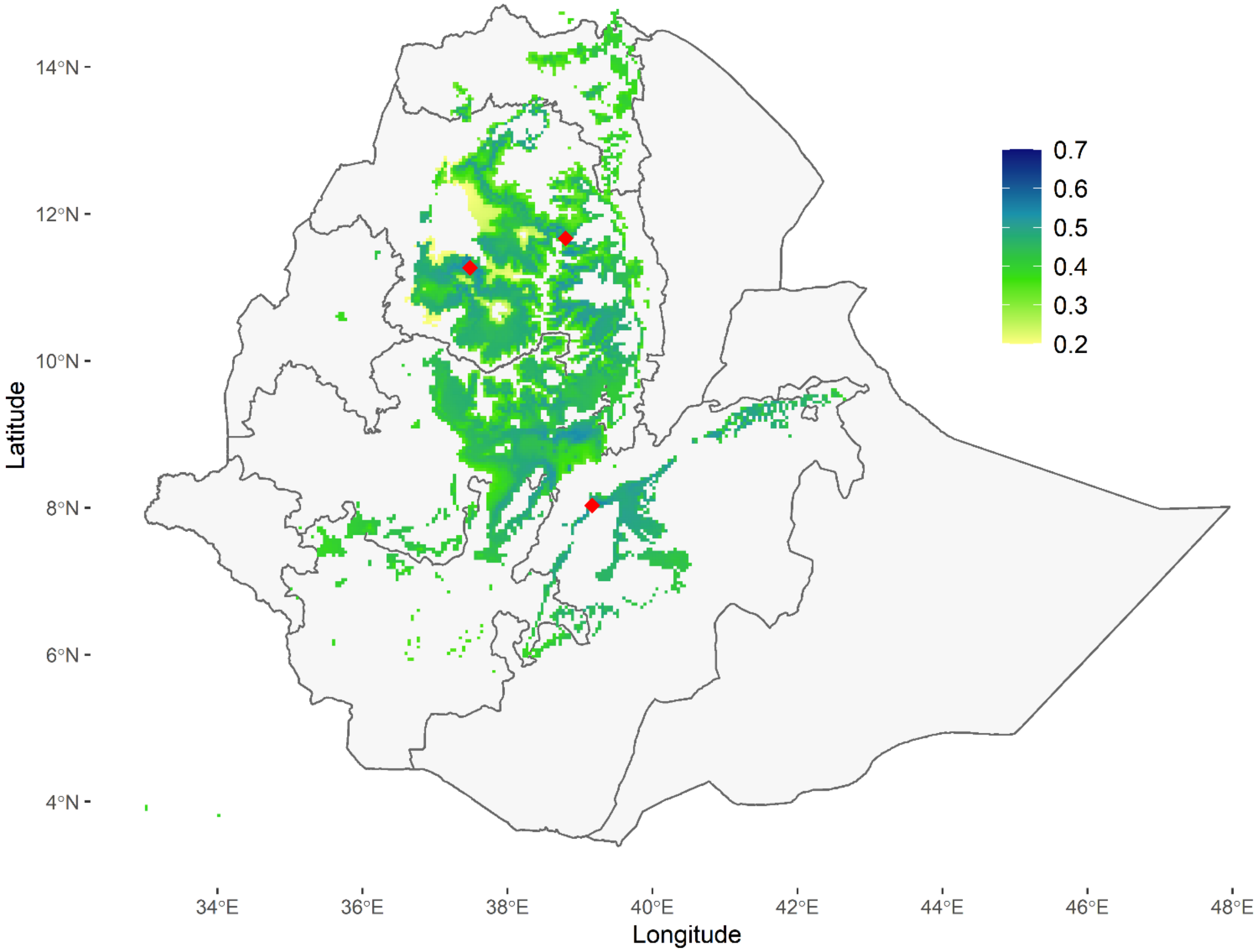

Fig. S4. Climate similarity between the experimental locations (in red) and the Ethiopian landscape. Similarity in shown in color from 0.2 to 0.7, the higher the value the more similar the place is to the station. Areas depicted in gray have negligible similarity to the experimental stations.

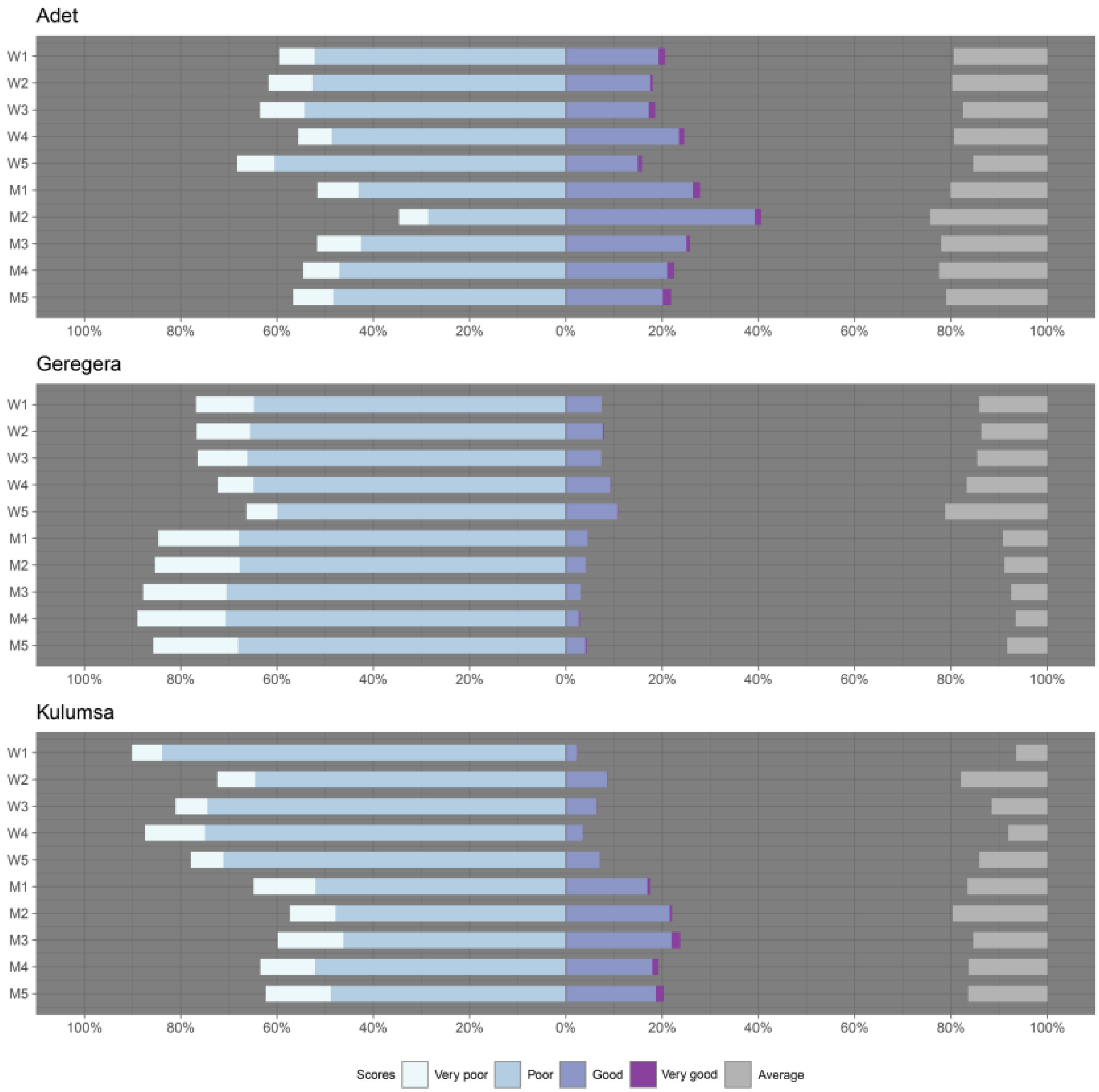

Fig. S5. Breakdown of Likert scores provided by farmers in each of the test locations, colored according to legend. Individual farmers' distributions are arranged on a common scale (on the x axis), with 0 centered between "Poor" (score 2) and "Good" (score 4). To the right, the "Average" (score 3) proportion is reported. Each row amounts to 100% of scores given by an individual man or woman farmer, reported as M1-M5 and W1-W5, respectively.

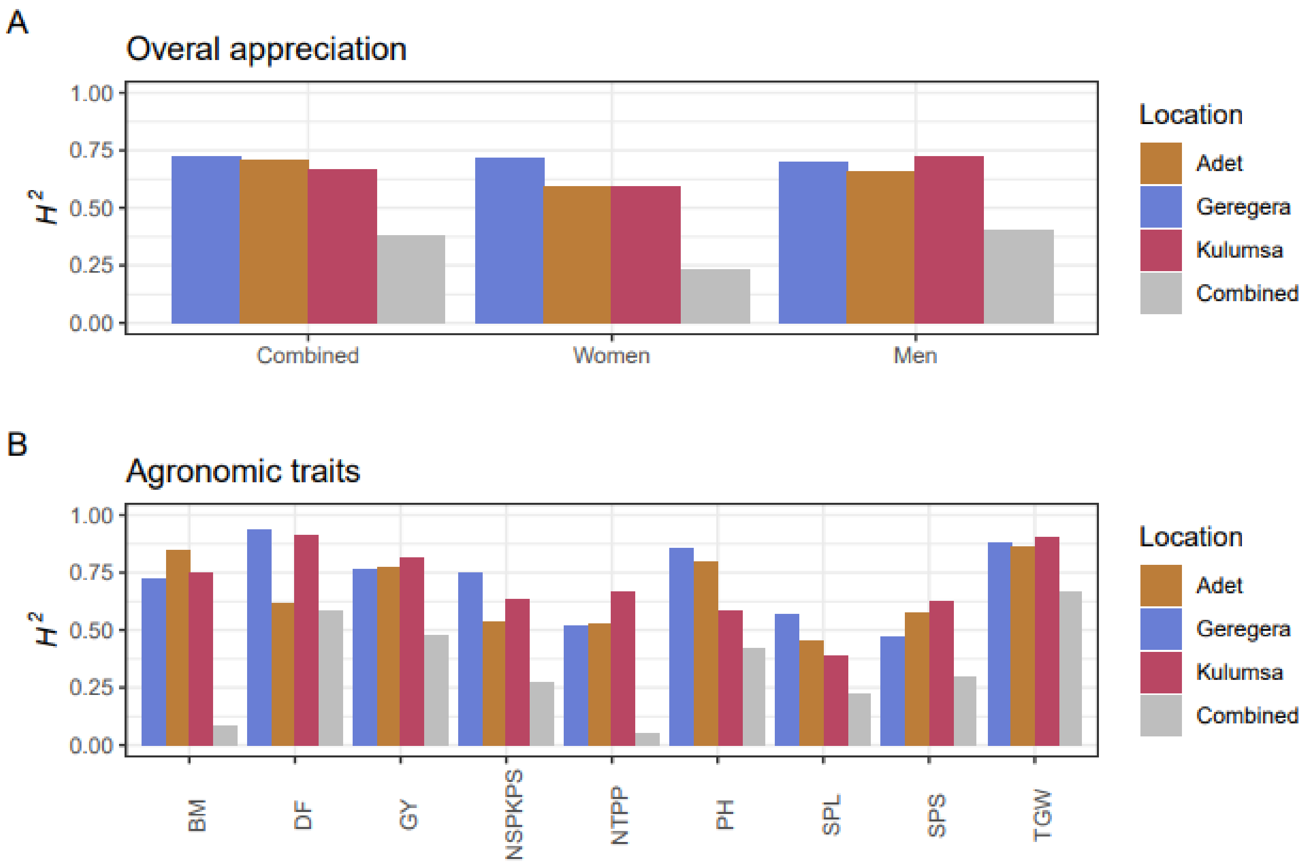

Fig. S6. Broad sense heritability (H^2) value estimates for (A) overall appreciation and (B) agronomic traits measured on the EtNAM, colored according to legend. BM, biomass; DF, days to flowering; GY, grain yield; NSPKPS, number of spikelets per spike; PH, plant height; SPL, spike length; SPS, seeds per spike; TGW, thousand grain weight.

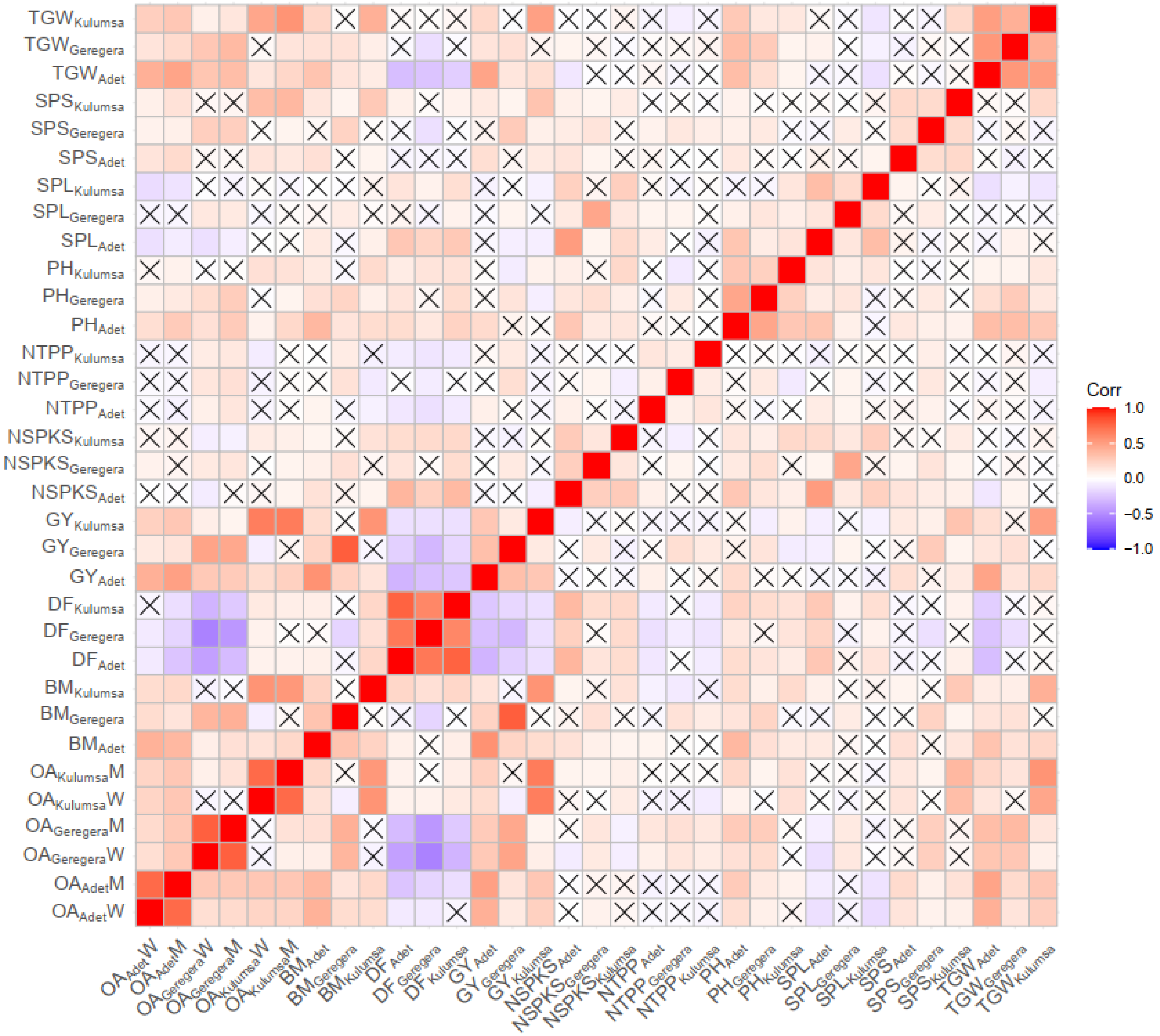

Fig. S7. A symmetric correlation plot reporting Pearson's correlation coefficient between and among EtNAM traits as BLUPs. OA evaluations and agronomic evaluations are divided by gender and by location, respectively. Direction and value of the correlation metric is given in colors according to legend. Crossed-out squares represent non-significant correlations ($p > 0.05$). OA, overall appreciation; BM, biomass; DF, days to flowering; GY, grain yield; NSPKS, number of spikelets per spike; NTPP, number of tillers per plant; PH, plant height; SPL, spike length; SPS, seeds per spike; TGW, thousand grain weight.

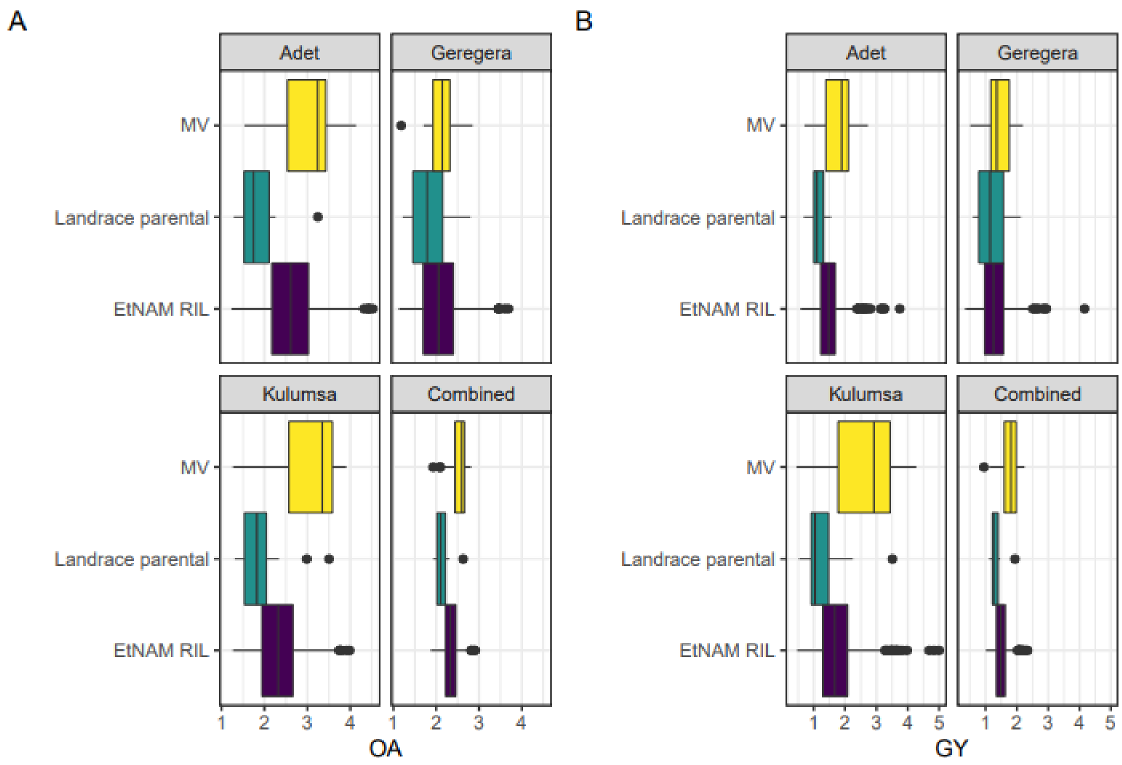

Fig. S8. Farmer choice distribution. A) Detail of overall appreciation (OA) and B) grain yield (GY) values by type of genetic materials, *e.g.* modern varieties (MV), landraces parentals to the EtNAM, and EtNAM RILs. On the x axis, the phenotypic BLUP value in either OA or GY units.

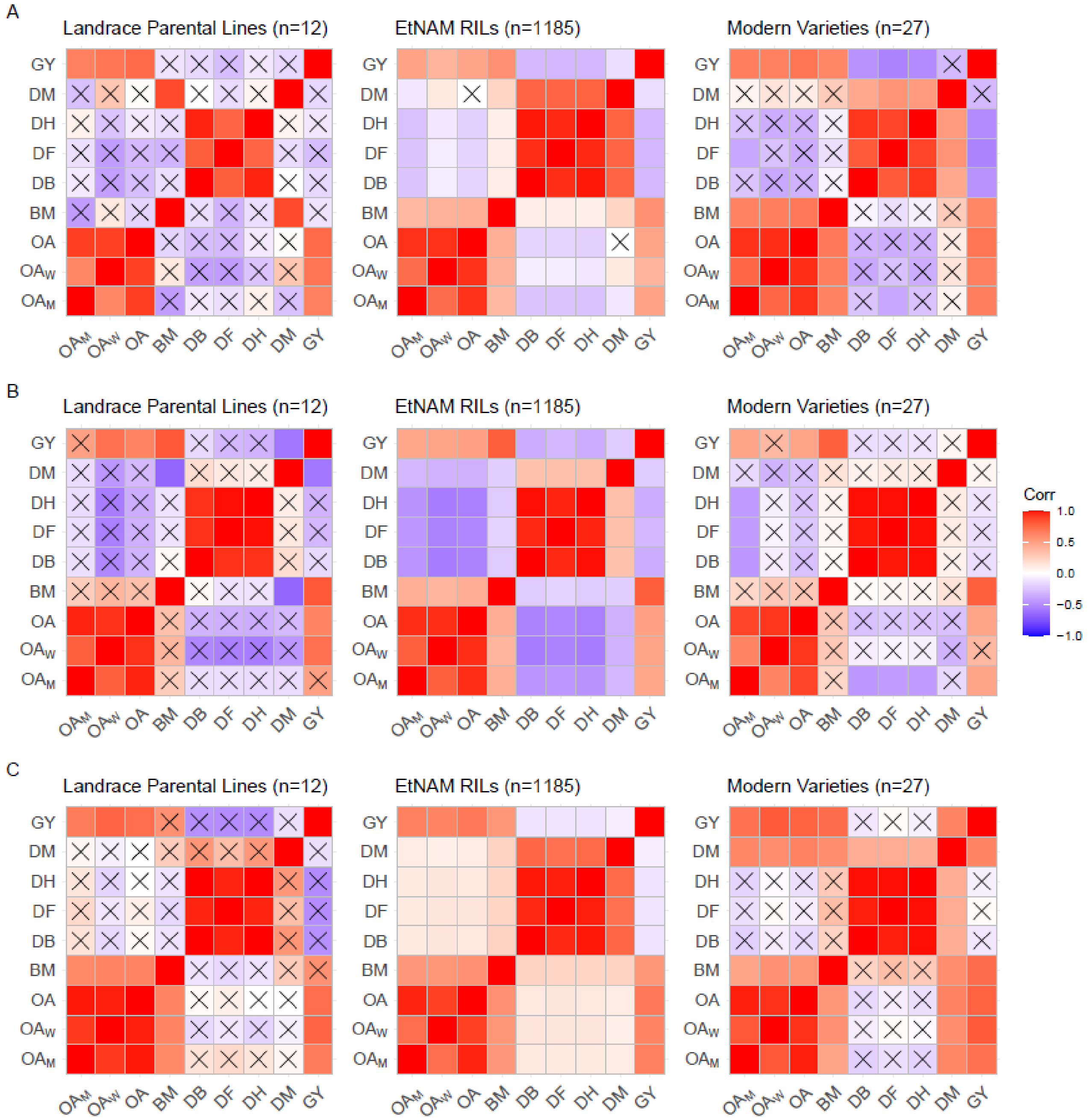

Fig. S9. A correlation plot representing Pearson's correlation coefficients between traits by type of genetic materials (Landraces parental lines of the EtNAM, EtNAM RILs, modern varieties) and by location (A= Adet, B=Geregera, C=Kulumsa). Direction and value of the correlation metric is given in colors according to legend. Crossed-out squares represent non-significant correlations ($p > 0.05$).

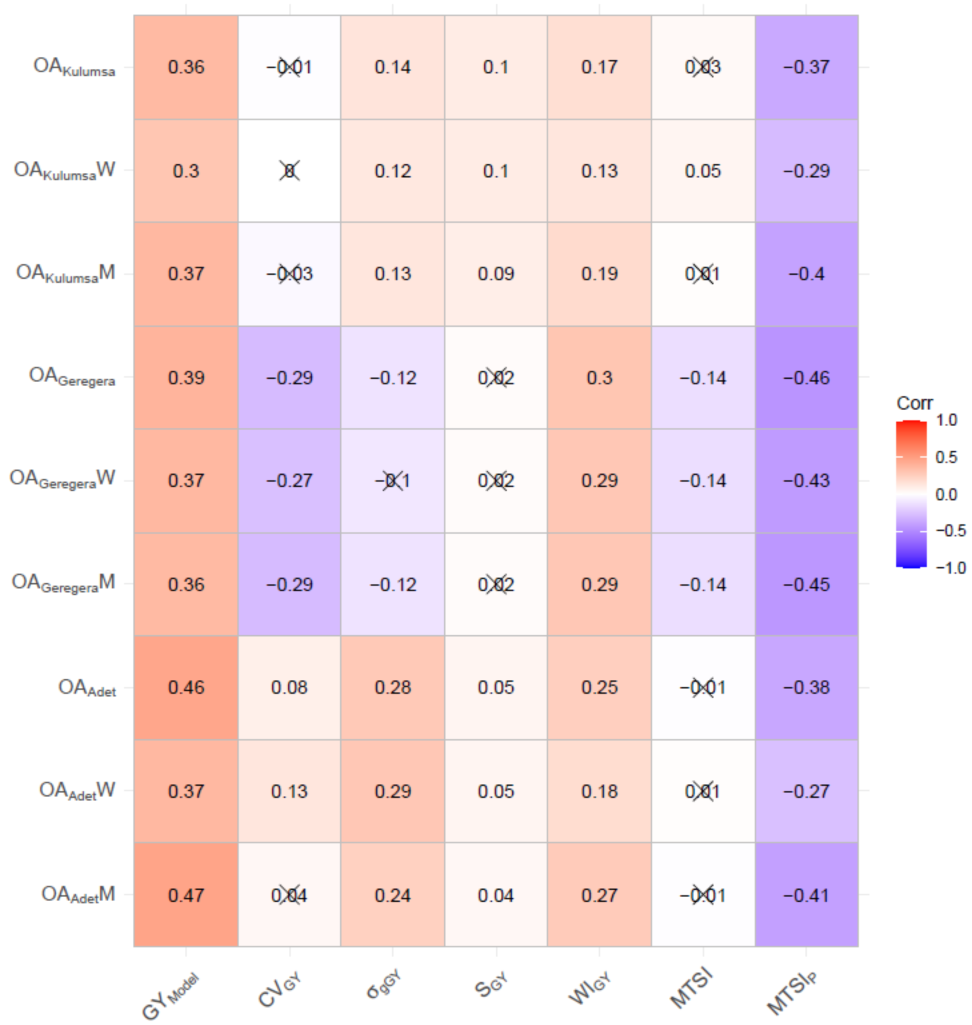

Fig. S10. A correlation plot representing Spearman correlation coefficients between OA evaluations given in each location (rows in the matrix) and stability metrics derived from the combination of the EtNAM test fields (columns in the matrix). Direction and value of the correlation metric is given in colors according to legend. Crossed-out squares represent non-significant correlations (p > 0.05). OA, overall appreciation, divided by gender and location; GY model, GY value derived from the stability model; CV_{GY}, coefficient of variation; σ_gGY, genotypic variance; S_{GY}, Shukla's stability index; WI_{GY}, Annicchiarico's genotypic confidence index; MTSI, multi-trait stability index, considering stability; $MTSI_P$, multi-trait stability index considering stability and genotype performance.

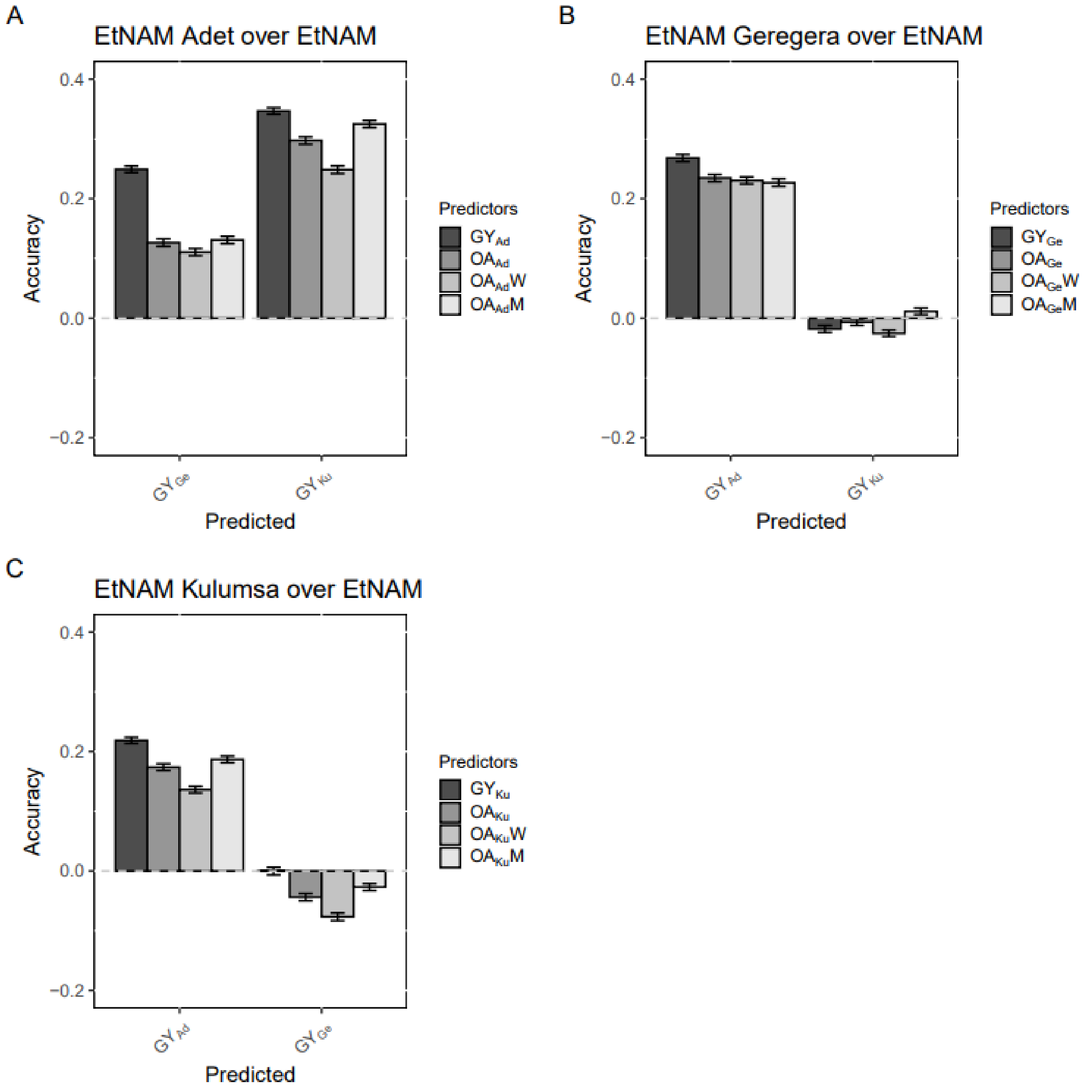

Fig. S11. Prediction accuracies of a GBLUP model trained on location-specific trait values of the EtNAM and used to predict EtNAM grain yield (GY) in other locations. (A) Prediction accuracy of GY, OA and OA by gender (W or M) measured in Adet to predict GY in Geregera and Kulumsa. The accuracy of the prediction is reported on the y axis with bars indicating SEM across 100 repetitions. The predictors are color coded according to legend, while predicted GY measures are reported on the x axis. (B) Prediction accuracy of Geregera data to predict GY in Adet and Kulumsa, represented as in panel A. (C) Prediction accuracy of Kulumsa data to predict GY in Adet and Geregera, represented as in panel A.

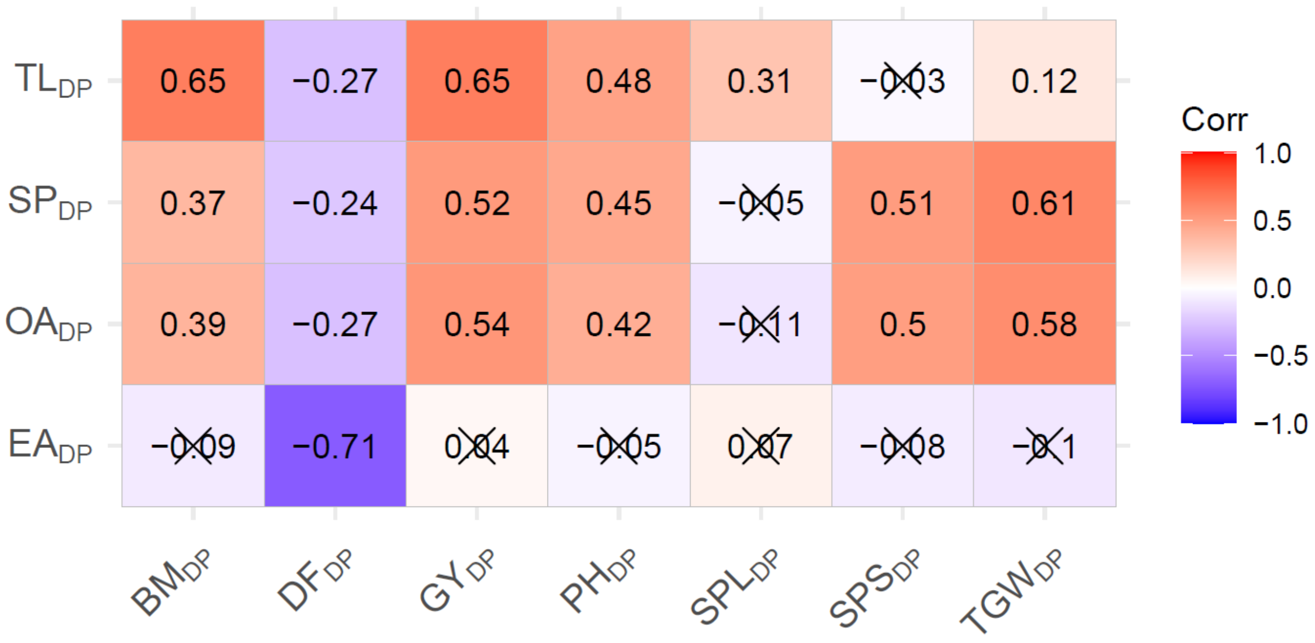

Fig. S12. A correlation plot representing Pearson correlation coefficients between PVS evaluations (rows in the matrix) and trait measures (columns in the matrix) on the DP. Direction and value of the correlation metric is given in colors according to legend. Crossed-out squares represent non-significant correlations ($p > 0.05$). PVS traits are: EA, earliness; OA, overall appreciation; SP, spike appreciation; TL, tillering capacity. Agronomic traits are: BM, biomass; DF, days to flowering; GY, grain yield; PH, plant height; SPL, spike length; SPS, seeds per spike; TGW, thousand grain weight.

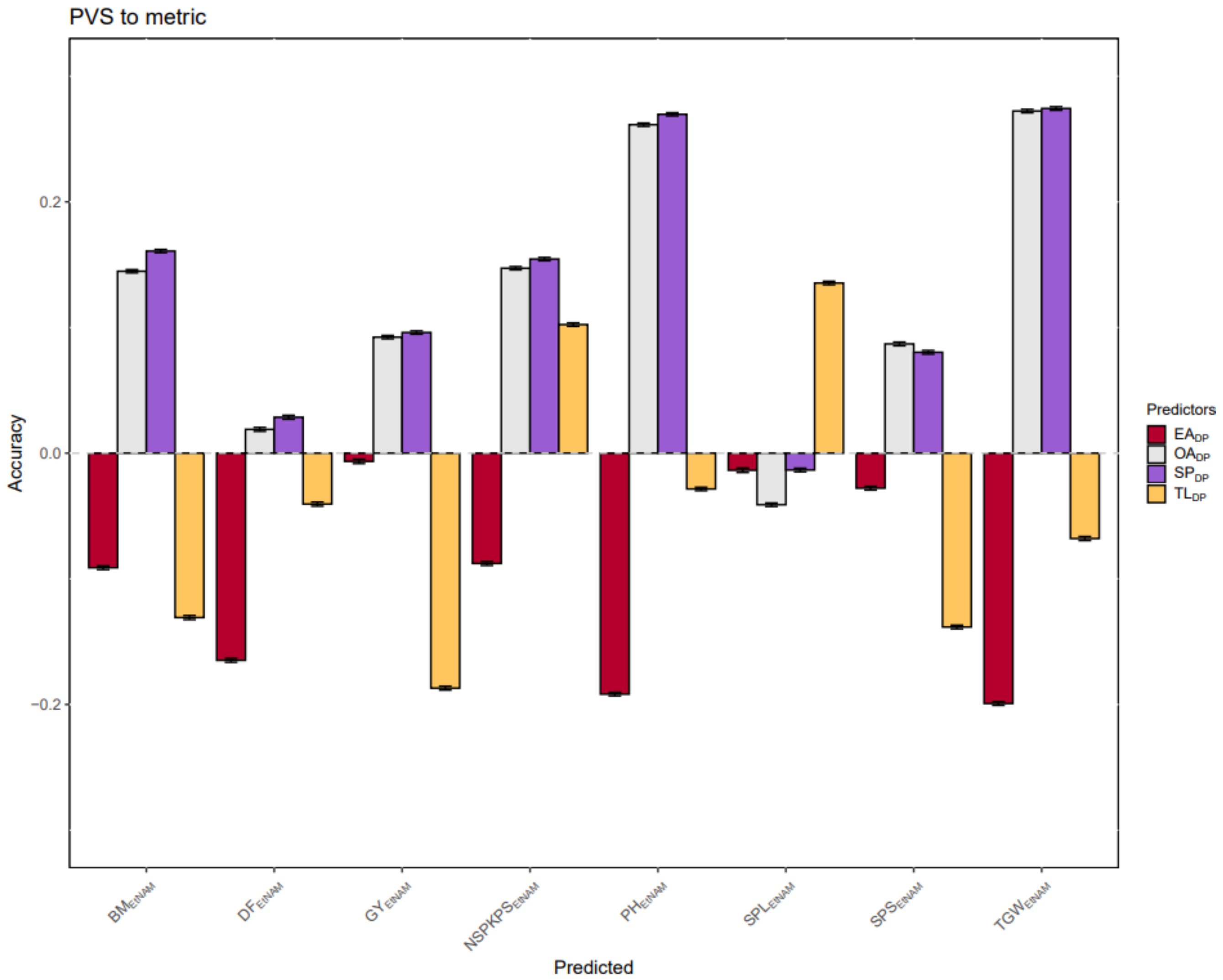

Fig. S13. Prediction accuracy of a model trained on PVS traits measured on the DP and tested on agronomic performance of the EtNAM. Predictor PVS traits are: EA, earliness; OA, overall appreciation; SP, spike appreciation; TL, tillering capacity. Predicted agronomic values are: BM, biomass; DF, days to flowering; GY, grain yield; NSPKPS, number of spikelets per spike; PH, plant height; SPL, spike length; SPS, seeds per spike; TGW, thousand grain weight.

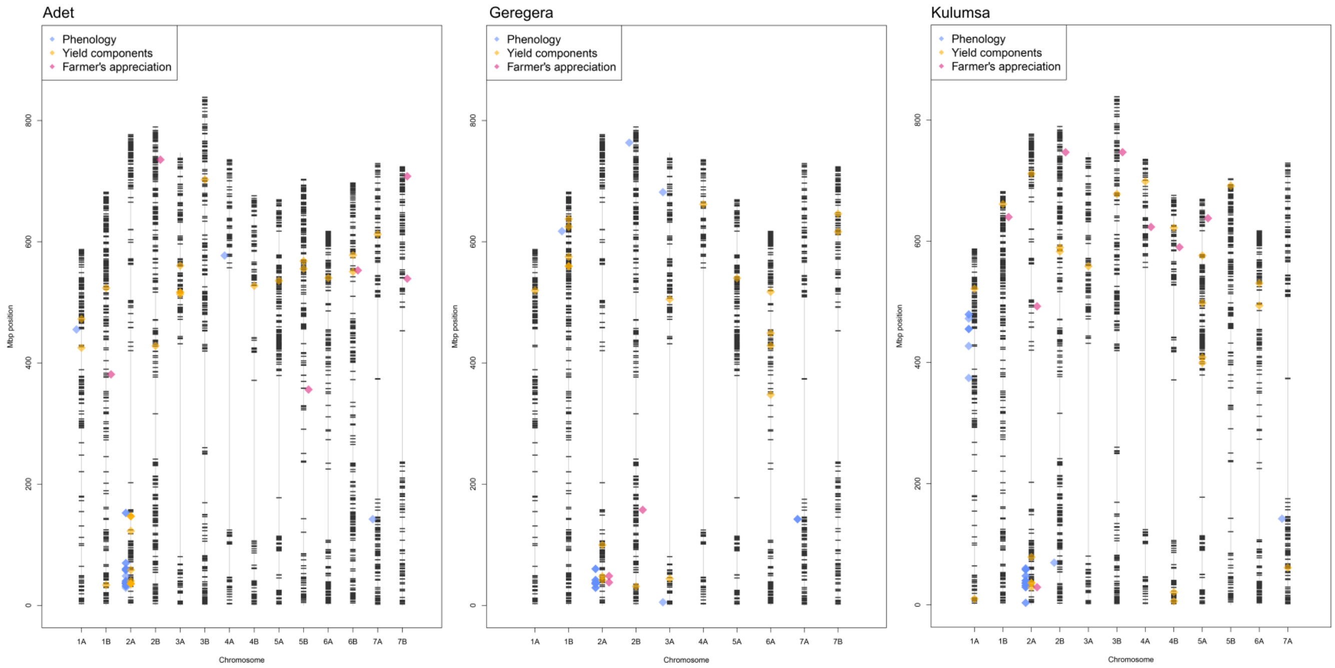

Fig. S14. QTL mapping on individual EtNAM families, by location. Markers included in EtNAM genetic maps are reported following their physical position as black ticks. Chromosomes with at least one detected QTL are shown. QTL are shown in colors according to legend, and correspond to phenotypes grouped by phenology (DB, DH, DF, DM), yield components (GY, TGW, SPL, NSPKPS, SPS, PH, NTPP, BM), farmers' appreciation (OA). QTL markers are semi-transparent and have deeper shades of color proportionally to the number of EtNAM subfamilies in which they are detected.
